# Cell Membrane Vesicles with Enriched CXCR4 Display Enhances Their Targeted Delivery as Drug Carriers to Inflammatory Sites

**DOI:** 10.1002/advs.202101562

**Published:** 2021-10-23

**Authors:** Dandan Wang, Shengjie Jiang, Fengyi Zhang, Siqin Ma, Boon Chin Heng, Yuanyuan Wang, Junxia Zhu, Mingming Xu, Ying He, Yan Wei, Xuehui Zhang, Bin Xia, Xuliang Deng

**Affiliations:** ^1^ Department of Pediatric Dentistry School and Hospital of Stomatology Peking University Beijing 100081 P. R. China; ^2^ Beijing Laboratory of Biomedical Materials Department of Geriatric Dentistry Peking University School and Hospital of Stomatology Beijing 100081 P. R. China; ^3^ Department of Dental Materials & Dental Medical Devices Testing Center National Engineering Laboratory for Digital and Material Technology of Stomatology Peking University School and Hospital of Stomatology Beijing 100081 P. R. China

**Keywords:** apical periodontitis, cell membrane vesicles, curcumin, CXCR4, inflammatory bowel disease

## Abstract

Cell membrane vesicles (CMVs) are composed of natural cell membranes which makes them effective drug delivery systems with low immunogenicity and prolonged circulation time. However, targeting delivery of CMVs in vivo for clinical applications is still a major challenge. In this study, CXCR4 recombinant lentivirus is transfected into MC‐3T3 cells and membrane CXCR4‐enriched MC‐3T3 cells are obtained. CMVs with enriched membrane CXCR4 display (CXCR4‐CMVs) are obtained from the transfected MC‐3T3 cells. Curcumin, an effective natural anti‐inflammatory compound, is encapsulated into CXCR4‐CMVs through physical entrapment (CXCR4/Cur‐CMVs), with the membrane integrity of CXCR4/Cur‐CMVs being well‐preserved. CXCR4/Cur‐CMVs induce enhanced M2 macrophage polarization, exhibit anti‐inflammatory effects, and significantly improve homing via the CXCR4/CXCL12 axis in vitro. Utilizing ulcerative colitis and apical periodontitis as inflammatory disease models, it is found that CXCR4/Cur‐CMVs are obviously aggregated within inflammatory areas after intravenous administration, which results in significant amelioration of ulcerative colitis and apical periodontitis. Therefore, this research may provide a feasible and innovative approach for fabricating an inflammatory site‐targeting delivery system, by engineering CMVs to increase membrane‐presenting CXCR4 receptor.

## Graphical abstract

Effective inflammation‐targeting delivery for clinical applications is still a challenge. Cell membrane vesicles without (CMVs) and with enriched CXCR4 display (CXCR4‐CMVs) are fabricated. CMVs retain natural cell membrane structure and composition, which confers lower immunogenicity, prolonged circulation time, and enhanced targeting capacity. Curcumin is encapsulated within CXCR4‐CMVs (CXCR4/Cur‐CMVs), and the anti‐inflammatory and inflammation‐targeting effects of CXCR4/Cur‐CMVs are characterized.

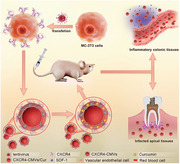

## 1 Introduction

A close connection exists between inflammation and various chronic diseases/disorders, including inflammatory bowel disease (IBD), autoimmune diseases, and cancer.^[^ ^1^, ^2^, ^3^ ^]^ To date, inflammation and inflammation‐related diseases remain an intractable and formidable challenge in clinical treatment. Despite numerous anti‐inflammation drugs being developed over the past few decades, drug overdose and systemic adverse reactions still pose major challenges.^[^ ^4^ ^]^ Thus, drug delivery systems have been extensively explored to improve their bioavailability and efficacy. Recently, cell membrane‐coated carriers have been explored for drug delivery by camouflaging particles to evade the immune system.^[^ ^5^, ^6^ ^]^ However, during the procedure of membrane coating, the membrane could be destroyed and their long‐term safety and stability still need further improvements.^[^ ^7^ ^]^ Extracellular vesicles (EVs), small membrane‐structured particles produced by almost all cell types, have been extensively utilized in various biomedical fields, such as therapeutic intervention and drug delivery.^[^ ^8^ ^]^ However, EVs are formed inside the cell and their membrane composition and structure are not exactly the same as the cell membrane. In particular, their compatibility and durability are still questionable and the yield of naturally produced EVs is still low.^[^ ^9^ ^]^

Recently, novel cell membrane vesicles (CMVs) have been produced that retain natural cell membrane structure and composition. These vesicles were induced by cytochalasin B (CB), a drug that modulates reversible cytoskeleton membrane interactions. The natural cell membrane confers CMVs with various advantages, including lower immunogenicity and cytotoxicity, prolonged circulation time, and enhanced targeting capacity after surface modification.^[^ ^10^, ^11^, ^12^ ^]^ Bioactive agents or drugs could be loaded into CMVs.^[^ ^11^, ^12^ ^]^ For example, curcumin, an effective natural anti‐inflammatory compound extracted from rhizomes of Curcuma longa,^[^ ^13^ ^]^ can be loaded into CMVs (Cur‐CMVs) to overcome its poor bioavailability.^[^ ^14^ ^]^ Various alternative cell sources, low cost of manufacture, simple fabrication method, and high preparation efficiency make CMVs promising vesicles for drug delivery. However, efficient and specific targeting delivery of CMVs to inflammatory sites in vivo remains a tricky challenge.

Development of specific inflammation site‐targeting CMVs delivery system is absolutely crucial for their clinical translation. Several different strategies have been employed for construction of targeting delivery system. For example, active targeting strategies focus on the conjugation of ligands, such as aptamers or peptides, to the surface of carriers.^[^ ^15^, ^16^, ^17^ ^]^ However, biochemical conjugation might alter the natural characteristics and functions of the engineered vesicles and complex conditions in vivo might weaken their targeting properties. Immune cells have also been developed as specific delivery systems due to their inherent targeting ability.^[^ ^18^, ^19^ ^]^ However, cell‐based drug delivery systems tend to be larger in size and not conducive to blood circulation in vivo. CXCL12, also known as stromal cell derived factor‐1 (SDF‐1), is a member of the CXC chemokine subfamily, which interacts specifically with the corresponding 7‐transmembrane domain G‐protein‐coupled receptor CXCR4. Previous studies have shown that CXCL12 levels increase markedly in inflammatory tissues and that CXCL12/CXCR4 binding plays an important role in chemotactic processes, such as leukocyte trafficking and recruitment.^[^ ^20^, ^21^, ^22^, ^23^ ^]^ Therefore, we hypothesize that CMVs with enriched expression of membrane receptor CXCR4 through genetic engineering of parental cells could be exploited as a targeting delivery strategy for treatment of inflammatory diseases.

**Figure 1:**
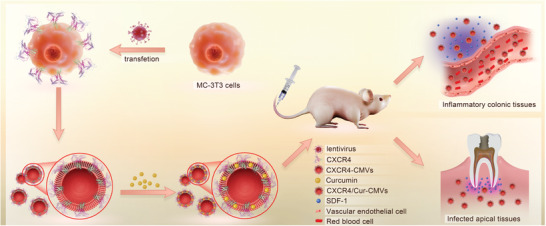
The schematic illustration of CXCR4‐CMVs fabrication, inflammation targeting, and modulation in vivo.

In this study, we recombinantly overexpressed CXCR4 in MC‐3T3 cells via lentivirus transfection, so as to obtain CMVs with enriched membrane CXCR4 (CXCR4‐CMVs). Then curcumin was encapsulated into CXCR4‐CMVs through physical entrapment (CXCR4/Cur‐CMVs). The fabricated CXCR4/Cur‐CMVs possessed natural membrane surface characteristics, together with stable display of the targeting protein CXCR4, thus achieving excellent biocompatibility and enhanced targeting properties (**Figure** **1**). Utilizing ulcerative colitis and apical periodontitis as disease models, we found that CXCR4/Cur‐CMVs could effectively target inflammation sites in vivo and significantly ameliorate the inflammatory symptoms of ulcerative colitis and apical periodontitis, thus suggesting that CMVs with enriched display of membrane CXCR4 might be a promising inflammation‐targeting delivery system for inflammatory disease treatment.

## 2 Results and Discussion

### 2.1 Preparation and Characterization of CXCR4/Cur‐CMVs

**Figure 2:**
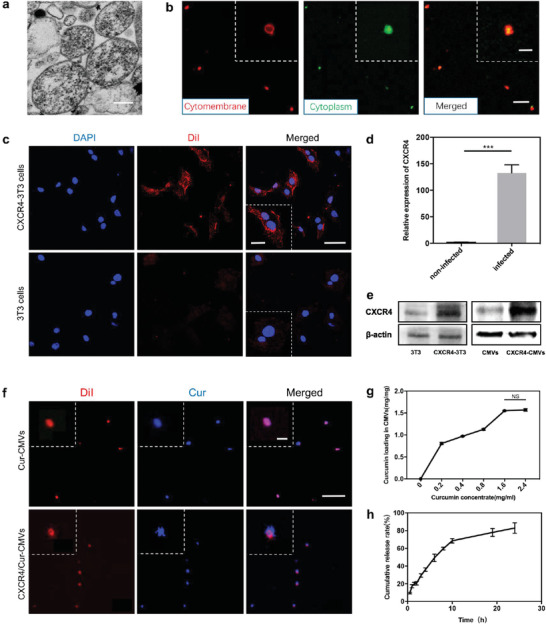
The characterization of CXCR4‐CMVs and encapsulation of curcumin into CXCR4‐CMVs. a) Representative TEM images of CMVs (the scale bar = 300 nm). b) Representative CLSM images of DiI (red) and cFDA‐SE (green) co‐labeled CMVs (the scale bar = 5 µm), with insets showing a higher magnification (the scale bar = 1 µm). c) Membrane CXCR4 protein fluorescence expression (red) in 3T3 cells of the transfection group were much higher, as compared to wild‐type 3T3 cells. 4′,6‐diamidino‐2‐phenylindole (DAPI)‐labeled cell nuclei are shown in blue (the scale bar = 50 µm) with inset showing a higher magnification (the scale bar = 20 µm). d) The CXCR4 mRNA level of infected cells was significantly higher than noninfected 3T3 cells (***p<0.001, *n* = 3). e) CXCR4 protein expression levels of CXCR4‐3T3 and CXCR4‐CMVs increased markedly after lentivirus transfection, as shown by western blot analysis. f) Representative CLSM images of CMVs and CXCR4‐CMVs encapsulated with curcumin; Curcumin auto‐fluorescence (blue), DiI‐labeled CMVs and CXCR4‐CMVs (red) images provided (the scale bar = 10 µm) with insets showing higher magnification (the scale bar = 1 µm). g) The curcumin loading effectiveness curve. h) The cumulative drug release curve of curcumin.

In this study, MC‐3T3 cells were selected to prepare CMVs due to several advantageous properties such as short mitotic cycle, immortalized lifespan, and being easily transfectable by lentivirus. After treatment with Cytochalasin B (CB), the morphological changes of the cells were dynamically observed under fluorescence confocal microscopy. The fibrous framework of the cells was shrunken and spherical cell capsules could be observed surrounding the cells (Figure S1, Supporting Information). After vortex and gradient centrifugation, the CMVs were separated from 3T3 cells. Transmission electron microscope (TEM) observation verified that the ultrastructure of the CMVs was intact and that the inner cytoplasmic content of the CMVs was wrapped by complete membrane structures (**Figure** **2**a), which are consistent with previous studies that the natural cell membrane and cytoplasm of parental cells are well‐preserved in CMVs.^[^ ^10^, ^11^, ^12^ ^]^ Next, CMVs were obtained from DiI/cFDA‐SE co‐labeled 3T3 cells, with the red fluorescence and green fluorescence of the CMVs localized on the membrane structure and cytosolic fluids, respectively, which validated that the membrane structure and the fluid content of CMVs were from the parental cells (Figure 2b). The average size of CMVs analyzed by dynamic light scattering (DLS) was ≈827.4 nm (Figure S2, Supporting Information). Raw264.7 cells were then used to test the biocompatibility of CMVs. CCK8 assay results showed that there were no significant differences between the groups, thus indicating that CMVs displayed insignificant cytotoxicity to immune cells (Figure S3, Supporting Information). Moreover, the CMVs could be stored for weeks without losing structural integrity.^[^ ^11^, ^12^ ^]^ All these data thus showed that natural‐membrane CMVs fabricated with a low‐cost, one‐step, and stable efficient protocol are different from previously reported EVs, which were generated inside cells through an intracellular procedure.

To improve the inflammation site‐targeting delivery of CMVs, we fabricated CMVs with enriched expression of membrane CXCR4 via lentivirus transfection of 3T3 cells. As mentioned above, CMVs preserved the original cell membrane structure, and we in turn fabricated CMVs from 3T3 cells with enriched expression of cell surface protein‐CXCR4. After transfection with lentivirus vector encoding the CXCR4/GFP chimeric protein, 3T3 cells exhibited markedly visible GFP fluorescent signals in the transfection group, as compared to wild‐type 3T3 cells, which suggests effective transfection (Figure S4, Supporting Information). The membrane CXCR4 protein fluorescence expression (red) in the 3T3 cells of the transfection group was much higher compared to wild‐type 3T3 cells (Figure 2c). Reverse transcription polymerase chain reaction (RT‐PCR) showed that the mRNA level of CXCR4 was significantly higher in the transfected group than the vehicle group (Figure 2d). The CXCR4 protein levels in 3T3 cells and CMVs of the transfection group were much higher compared to wild‐type 3T3 cells and their corresponding CMVs, respectively, as shown by western blot analysis (Figure 2e). The TEM image showed that CMVs enriched with CXCR4 display maintained membrane integrity (Figure S5, Supporting Information). Besides, there was no difference in Zeta potential between CXCR4‐CMVs and CMVs (Figure S6, Supporting Information). Collectively, the results demonstrated that CXCR4 overexpression led to enriched membrane CXCR4 expression in 3T3 cells and that CMVs obtained from CXCR4‐overexpressing 3T3 cells displayed enriched membrane CXCR4, because the natural membrane structure is well‐preserved.

Curcumin, a natural small molecule product extracted from turmeric, shows strong anti‐inflammatory properties and numerous studies indicated that curcumin may be a potential therapeutic agent in many chronic inflammatory diseases such as inflammatory bowel disease, arthritis, and pancreatitis.^[^ ^13^, ^24^ ^]^ Curcumin can be easily loaded into lipid bilayers by physical entrapment due to its lipophilicity.^[^ ^25^, ^26^ ^]^ Thus, we speculated that curcumin could be loaded into CMVs through interaction between the hydrophobic tails and hydrophobic drug. Fluorescence images showed an overlay of red fluorescent CXCR4‐CMVs with blue fluorescent curcumin, thus confirming that curcumin was successfully incorporated into CXCR4‐CMVs (Figure 2f and Figure S7, Supporting Information).

Curcumin often exhibits poor bioavailability because of its insolubility in water, rapid metabolism, and systemic elimination.^[^ ^27^ ^]^ In our study, we found that free curcumin in phosphate buffered saline (PBS) degraded easily and only 30% remained in the solution, while ≈90% of curcumin loaded into CMVs remained after 150 min (Figure S8a, Supporting Information). The yellow color of free curcumin in PBS rapidly lightened while the color of CXCR4/Cur‐CMVs in PBS barely changed within 2.5 h (Figure S8b, Supporting Information), indicating that curcumin encapsulated into CXCR4‐CMVs exhibited more stability through interaction of curcumin with the lipid bilayer. The curcumin loading efficiency was then investigated and the results showed that the binding capacity did not increase further when the concentration of curcumin reached to 1.6 mg mL^−1^. Thus the binding capacity was estimated to be ≈1.57 g curcumin to 1 g CMVs (Figure 2g and Figure S9, Supporting Information). Next, we tested the cumulative release rate of curcumin after dialysis for 24 h. The results showed that about 68% of curcumin was released within the first 10 h, and the release rate was up to about 83% within 24 h (Figure 2h), thus demonstrating that the anti‐inflammatory effects of curcumin lasted for at least 24 h, and that CMVs could be exploited as a drug‐sustained release carrier.

TEM images of Cur‐CMVs and CXCR4/Cur‐CMVs showed intact membrane and cytoplasmic structure, which were similar to CMVs and CXCR4‐CMVs (Figure S10, Supporting Information). Moreover, the CMVs, CXCR4‐CMVs, Cur‐CMVs, and CXCR4/Cur‐CMVs had similar zeta potentials (Figure S6, Supporting Information).

### 2.2 Evaluation of Anti‐Inflammatory and Targeting Ability of CXCR4/Cur‐CMVs via the CXCL12/CXCR4 Signaling Axis In Vitro

**Figure 3:**
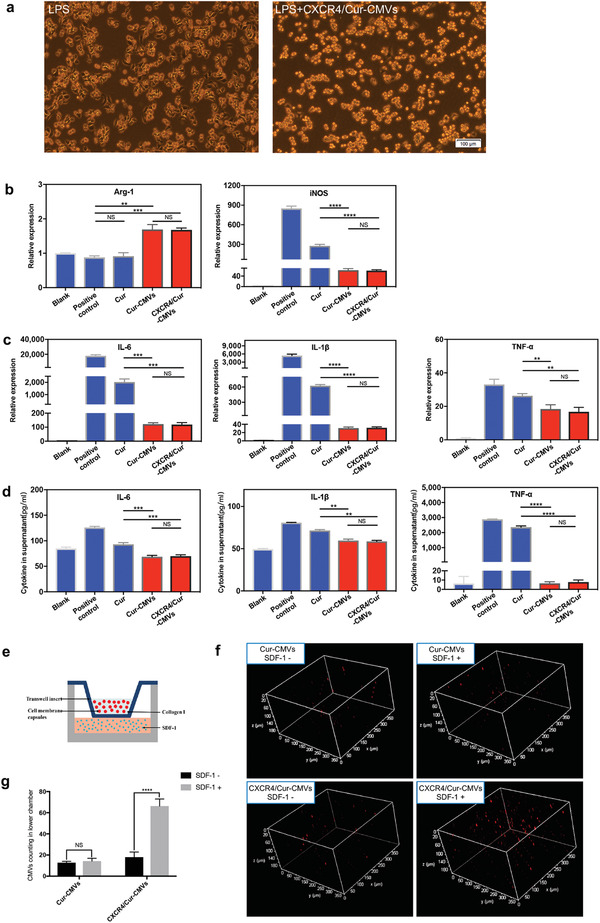
The anti‐inflammation activity of CXCR4/Cur‐CMVs and the targeting capacity of CXCR4/Cur‐CMVs via the CXCR4/CXCL12 signaling axis in vitro. a) Effects on the morphology of polarized macrophages after different treatments in the two groups. Macrophages in the LPS group were induced with LPS for 6 h; while macrophages in the LPS plus CXCR4/Cur‐CMVs group were treated with CXCR4/Cur‐CMVs for 1 h before induction with LPS for another 6 h. Macrophages in the LPS plus CXCR4/Cur‐CMVs groups showed round shape morphology, whereas apparent “synapses” of macrophage were exhibited in the LPS groups. b) Effects of different treatments on gene expression levels of M1 and M2 phenotype markers. c) Gene expression levels and d) secreted protein expression levels within the supernatant of IL‐6, IL‐1*β*, and TNF‐*α* by Raw264.7 cells, after different treatments in vitro. The expression levels of inflammatory cytokines within the supernatant were determined using an enzyme‐linked immunosorbent assay (**p<0.01, ***p<0.001, ****p<0.0001, *n* = 3). e) Schematic illustration of transwell chamber. f) Representative CLSM images of DiI‐labeled Cur‐CMVs and CXCR4/Cur‐CMVs collected in the lower chamber. The addition of CXCL12 significantly increased migration of CXCR4/Cur‐CMVs to the lower chamber, which had no significant effects on Cur‐CMVs migration. g) Counting of Cur‐CMVs and CXCR4/Cur‐CMVs in the lower chamber with and without CXCL12. The statistical results represent an approximately threefold difference in the amount of CXCR4‐CMVs after the addition of CXCL12 into the lower chamber (****p<0.0001, *n* = 10).

Classically activated macrophages (M1 macrophages) mainly mediate pro‐inflammatory processes, while alternatively activated macrophages (M2 macrophages) participate in anti‐inflammatory responses.^[^ ^28^ ^]^ M2 macrophages are round while M1 macrophages have more “synapses.”^[^ ^29^ ^]^ In our study, Raw264.7 cells were pretreated with free curcumin, Cur‐CMVs, and CXCR4/Cur‐CMVs, respectively, before treatment with lipopolysaccharide (LPS). Cells treated with only LPS were set as the positive control group. Untreated cells were set as the blank group. We found that macrophages displayed round shape in the Cur‐CMVs and CXCR4/Cur‐CMVs groups after treatment with LPS, similar to that in the blank group. Apparent “synapses” of macrophage was exhibited in the LPS group and LPS plus free curcumin group (**Figure** **3**a and Figure S11, Supporting Information). iNOS and Arg‐1 are the main markers of M1 and M2 macrophages, respectively.^[^ ^28^ ^]^ We found that CXCR4/Cur‐CMVs upregulated Arg‐1 expression and downregulated iNOS expression more effectively than free curcumin (Figure 3b). Collectively, these results indicate that curcumin loaded into CMVs and CXCR4/CMVs facilitated M2 macrophage polarization, which could play a key role in anti‐inflammatory treatment. These results were in accordance with our previous results, which showed that encapsulated curcumin possessed higher stability and more favorable bioavailability, as compared to free curcumin.

We next explored the effects of CXCR4/Cur‐CMVs on inflammatory cytokine secretion in LPS‐induced macrophages. We found that treatment with CXCR4/Cur‐CMVs and Cur‐CMVs led to lower mRNA levels of IL‐6, IL‐1*β*, and TNF‐*α* than free curcumin. Moreover, mRNA levels of IL‐6, IL‐1*β*, and TNF‐*α* were most highly expressed in the positive control group (Figure 3c). Consistently, protein contents of IL‐6, IL‐1*β*, and TNF‐*α* in the supernatant of Raw264.7 cells were lower in the Cur‐CMVs and CXCR4/Cur‐CMVs treatment groups, as compared to the free curcumin and positive control groups (Figure 3d). These results thus confirmed the pronounced anti‐inflammatory effects of Cur‐CMVs and CXCR4/Cur‐CMVs, due to the high stability and favorable bioavailability of curcumin loaded in CMVs and CXCR4/CMVs. Collectively, these results demonstrated that curcumin loading in CMVs could provide a new powerful anti‐inflammatory drug delivery system.

Curcumin is known to possess broad‐spectrum anti‐bacterial properties, and has been demonstrated to inhibit bacterial virulence factors, inhibit bacterial biofilm formation, and prevent bacterial adhesion.^[^ ^30^ ^]^ Although periapical infections are characterized by a complex microbiome, the most frequently isolated bacterium is *Enterococcus faecalis*.^[^ ^31^ ^]^ The results of antibacterial testing in vitro showed that curcumin loaded into CMVs and CXCR4‐CMVs inhibited the growth of *E. faecalis* more effectively than free curcumin (Figure S12, Supporting Information), thus suggesting that curcumin displayed antimicrobial activity against *E. faecalis*, and that curcumin loaded into CMVs and CXCR4‐CMVs exhibited greater antibacterial activity than free curcumin.

Next, we conducted transwell migration assays to examine the targeting capacity of CXCR4/Cur‐CMVs in response to CXCL12 in vitro (Figure 3e). Results showed that the addition of CXCL12 in the lower chamber significantly elevated the DiI‐labeled CXCR4/Cur‐CMVs counts within the lower chamber, as compared with the group without addition of CXCL12 (Figure 3f). However, there was no significant difference of Cur‐CMVs counts in the lower chamber between the CXCL12 group versus the group without CXCL12 (Figure 3f,g), indicating that the addition of CXCL12 significantly increased migration of CXCR4/Cur‐CMVs to the lower chamber, while it had no significant effect on Cur‐CMVs migration. The statistical results showed an approximately threefold difference in the amount of CXCR4/Cur‐CMVs after the addition of CXCL12 into the lower chamber (Figure 3g). These results thus suggest that the engineered CMVs with enriched membrane CXCR4 expression displayed pronounced targeting response toward CXCL12 in vitro.

### 2.3 CXCR4/Cur‐CMVs Effectively Alleviated Ulcerative Colitis in Mice

**Figure 4:**
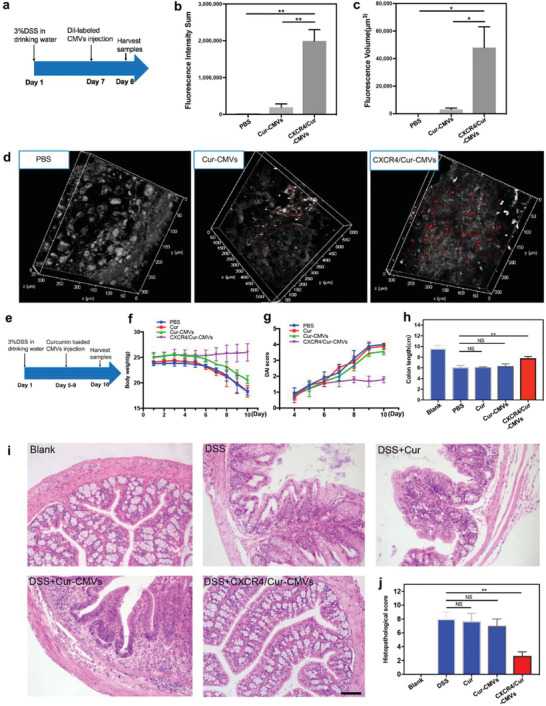
The inflammation modulation activity of CXCR4/Cur‐CMVs in the DSS‐induced ulcerative colitis model. a) Schematic illustration of inflammation‐targeting verification in the DSS‐induced ulcerative colitis model. b) Fluorescence volume and c) fluorescence intensity of the CXCR4‐CMVs group were much higher than the other two groups (*p<0.05, **p<0.01, *n* = 6). d) The intestinal tissues of colitis mice injected with CXCR4/Cur‐CMVs showed higher fluorescence expression, as compared to colitis mice injected with Cur‐CMVs in the 3D images (*n* = 6). e) Schematic illustration of inflammation modulation in the DSS‐induced ulcerative colitis model. Changes in f) body weight and g) DAI core of mice following different treatments: i) Control group (PBS injected only), ii) free curcumin group, iii) Cur‐CMVs group, iv) CXCR4/Cur‐CMVs group (*n* = 6). h) Colon length (cm) in different groups. Representative images of H&E staining of i) colonic tissues and j) histopathological scoring (**p<0.01, *n* = 6). The scale bar = 50 µm.

We constructed an acute inflammatory animal model of ulcerative colitis through dextran sulfate sodium (DSS) induction to verify the targeting and anti‐inflammation activity of CXCR4/Cur‐CMVs. DiI‐labeled CXCR4/Cur‐CMVs and Cur‐CMVs were injected intravenously on the 7th day after induction of colitis (**Figure** **4**a). Mice with ulcerative colitis injected with PBS were set as the control group. There was obvious red fluorescence staining of the colonic tissue within 24 h after CXCR4/Cur‐CMVs injection, while only very weak red fluorescent signal was observed with Cur‐CMVs (Figure 4d and Figures S13 and S14a, Supporting Information). The fluorescence intensity (Figure 4b) and volume (Figure 4c) were significantly higher in the CXCR4/Cur‐CMVs versus the Cur‐CMVs and control groups. As it was previously mentioned that CXCL12 is highly expressed in the inflammatory area, these results would imply that the CXCR/CXCL12 chemotactic axis plays an important role in CXCR4/Cur‐CMVs homing to areas highly expressing CXCL12, and that enriched membrane CXCR4 expression enhanced the migration and aggregation of engineered CMVs toward inflammatory sites with high expression of CXCL12. We also compared the inflammation‐targeting ability of CXCR4‐CMVs and CXCR4/Cur‐CMVs in vivo. The fluorescence imaging showed similar fluorescence signal distribution in the two groups, with no significant difference in fluorescence intensities between the two groups, indicating that encapsulation of curcumin might not influence the inflammation‐targeting ability of CXCR4‐CMVs (Figure S14, Supporting Information).

We then evaluated the anti‐inflammatory effects of CXCR4/Cur‐CMVs on ulcerative colitis in vivo. We administrated free curcumin, Cur‐CMVs and CXCR4/Cur‐CMVs into mice with ulcerative colitis for 5 consecutive days via intravenous injection (Figure 4e). Mice with ulcerative colitis injected with PBS were set as the control group, while mice without ulcerative colitis were set as the blank group. The results showed that mice with ulcerative colitis displayed intestinal inflammation, weight loss, diarrhea or bleeding, and colon shortening. In contrast, mice in the CXCR4/Cur‐CMVs injection group maintained a stable weight with a lower DAI score during the entire experimental duration, as compared with the control, curcumin, and Cur‐CMVs groups (Figure 4f,g), thus implying that CXCR4/Cur‐CMVs injection could relieve weight loss and alleviate severe diarrhea and fecal blood. Moreover, the colon length in the CXCR4/Cur‐CMVs group was only slightly shorter than the blank group, whereas the mice in the control, free curcumin, and Cur‐CMVs injection groups had drastically shortened colon length (Figure 4h and Figure S15, Supporting Information). We next further investigated whether CXCR4/Cur‐CMVs could alleviate the colonic inflammation histologically. Massive ulcers, epithelia defect, glandular damage, and inflammatory cell infiltration were observed in the control, free curcumin, and Cur‐CMVs groups, whereas mice in the CXCR4/Cur‐CMVs group displayed less severe inflammatory symptoms (Figure 4i), and its histological score was significantly lower than the control, free curcumin, and Cur‐CMVs groups (Figure 4j). Previous findings reported that the imbalance of pro‐inflammatory and anti‐inflammatory cytokines play an important role in the occurrence and development of IBD.^[^ ^32^ ^]^ Our results showed that the mRNA levels of IL‐6 and IL‐1*β* within the colonic tissues of the CXCR4/Cur‐CMVs group were significantly lower than those in the control, curcumin, and Cur‐CMVs groups, and only slightly higher than the blank group (Figure S16, Supporting Information). Collectively, these results demonstrate that CXCR4/Cur‐CMVs injection can alleviate colonic inflammatory response.

We analyzed the biodistribution of CXCR4/Cur‐CMVs and Cur‐CMVs in vivo and the results showed almost no fluorescence signal being observed in brain, heart, and kidney tissues. By contrast, fluorescent signals were observed in the spleen, lung, and liver in both the Cur‐CMVs and CXCR4/Cur‐CMVs groups (Figure S17, Supporting Information). The possible reasons might be DSS‐induced inflammation in the spleen and accumulation of large‐sized aggregated CMVs in the reticuloendothelial system such as lung and liver. Importantly, the fluorescence intensities in the major organs were lower than that of the targeted CMVs in the colonic tissues, indicating that CXCR4/Cur‐CMVs have inflammation‐targeting effects in vivo. We also evaluated systemic toxicity within the animal model through histological examination of major organs. The results showed little inflammatory cell infiltration within the spleen of the PBS group due to DSS‐induced inflammation, while in the other groups there was no obvious damage to major organs, indicating the compatibility of Cur‐CMVs and CXCR4/Cur‐CMVs in vivo (Figure S18, Supporting Information).

### 2.4 CXCR4/Cur‐CMVs Effectively Elicited Anti‐Inflammatory Reactions in Apical Periodontitis Areas

**Figure 5:**
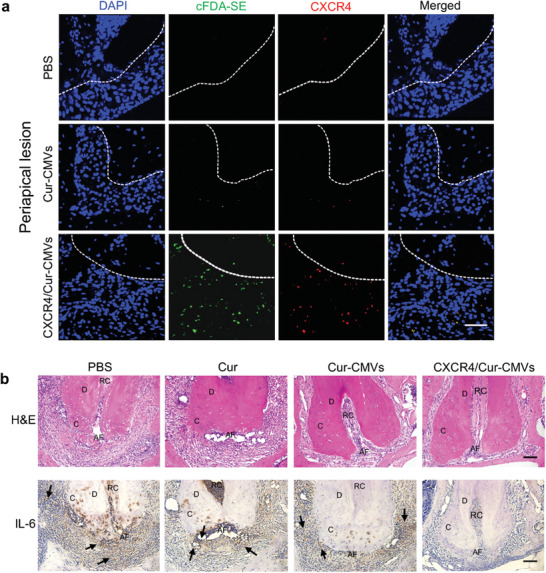
The anti‐inflammation activity of CXCR4/Cur‐CMVs in the apical periodontitis model. a) Cur‐CMVs, CXCR4/Cur‐CMVs recruitment, and CXCR4 expression in periapical lesions. Fluorescence images indicate that cFDA‐SE (green) labeled CXCR4/Cur‐CMVs were located in infected periapical tissues and co‐localized with the CXCR4 (red) at 24 h after injection. DAPI‐labeled cell nuclei are shown in blue. White dotted lines separate the periapical tissues and tooth root (*n* = 6). The scale bar = 50 µm. b) Inflammation regulation of CXCR4/Cur‐CMVs in an apical periodontitis model. Representative images of H&E staining and immunohistochemical staining of IL‐6 within the mandibular first molar in mice subjected to different treatments: i) Control group (PBS injected only), ii) free curcumin group, iii) Cur‐CMVs group, and iv) CXCR4/Cur‐CMVs group. The D denotes dentin; the C denotes cementum; the AF denotes apical foramen; the RC denotes root canal. The black arrows mark the immunohistochemical staining positive areas (*n* = 6). Scale bars = 200 µm.

In apical periodontitis (AP), the inflammation is difficult to control and systemic administration could lead to various detrimental side effects and low bioavailability. Hence, we established an apical periodontitis animal model as previously described^[^ ^33^ ^]^ and investigated whether CXCR4/Cur‐CMVs could exert effective anti‐inflammatory effects in AP. After pulp exposure for 21 days, the images of micro‐CT, hematoxylin and eosin (H&E), and immunohistochemistry showed obvious apical bone destruction, inflammatory cell infiltration, and increased secretion of inflammatory cytokines such as IL‐6, IL‐1*β*, and TNF‐*α*, thus confirming the successful construction of the AP model (Figure S19, Supporting Information). CXCL12, the most effective chemoattractant in stem cells homing,^[^ ^34^ ^]^ was found to be markedly upregulated within infected apical tissues (Figure S20a, Supporting Information). At 24 h after injection of the engineered CXCR4/Cur‐CMVs, cFDA‐SE labeled CXCR4/Cur‐CMVs aggregated within the corresponding areas of the apical lesions, while there was hardly any fluorescence signal in normal periapical tissues (Figure S20a,b, Supporting Information). We also found the co‐localization of highly expressed CXCR4 and cFDA‐SE‐labeled CXCR4/Cur‐CMVs in periodontitis apical tissues (**Figure** **5**a), while hardly any fluorescent signals could be detected in normal periapical tissues (Figure S21, Supporting Information). These findings thus confirm that increased CXCL12 expression in the periapical periodontitis area activates the recruitment of engineered CXCR4/Cur‐CMVs via the CXCR4/CXCL12 axis.

We then further investigated the anti‐inflammatory effects of CXCR4/Cur‐CMVs on AP in vivo. Mice with AP were injected with free curcumin, Cur‐CMVs, and CXCR4/Cur‐ CMVs, and mice with AP injected with PBS were set as the control group. H&E and immunohistochemistry staining images showed that after injection of CXCR4/Cur‐CMVs, the aggregation of inflammatory cells and secretion of IL‐6, IL‐1*β*, and TNF‐*α* in infected apical tissues markedly decreased compared with the control, free curcumin, and Cur‐CMVs injection groups (Figure 5b and Figures S22 and S23, Supporting Information), thus demonstrating that CXCR4/Cur‐CMVs effectively reduced the inflammatory response in infected apical tissues. Runx2, and the expression of early osteogenic markers in apical tissues, were upregulated in the CXCR4/Cur‐CMVs group to some extent (Figure S24, Supporting Information), suggesting anti‐inflammatory effects and early osteogenesis promotion within the apical periodontitis area after CXCR4/Cur‐CMVs systemic administration.

## 3 Conclusion

In our study, we have successfully fabricated engineered cell membrane capsules as a drug delivery carrier for specific targeting of inflammatory sites. CMVs derived from MC‐3T3 cells have natural structures and good biocompatibility. Next, we developed CMVs with enriched membrane CXCR4 display from MC‐3T3 cells transfected with CXCR4‐encoding lentiviral vectors. The engineered CMVs target inflammatory tissues through the CXCL12/CXCR4 chemotactic axis. CXCR4‐CMVs loaded with curcumin could maintain drug stability and activity, suppress the inflammatory reaction in vitro and demonstrate selective targeting and anti‐inflammatory effects on IBD and AP animal models. Our findings thus suggest that curcumin‐loaded CMVs of enriched membrane CXCR4 display could be a novel therapeutic agent for inflammatory disease treatment.

## 4 Experimental Section

### Cell Culture

Mouse MC‐3T3 cells and Raw264.7 cells were purchased from Cyagen Biosciences Inc. (USA), and cultured with complete *α*‐MEM (minimum essential medium) and Dulbecco's modified Eagle medium media (Gibco, Thermo Fisher Scientific, USA) respectively, supplemented with 10% (v/v) fetal bovine serum (FBS, Gibco, Thermo Fisher Scientific, USA) and 1% (v/v) penicillin‐streptomycin (Gibco, Thermo Fisher Scientific, USA) at 37 °C, within a humidified 5% CO_2_ incubator.

### Preparation of CMVs

MC‐3T3 cell cultures were utilized upon reaching 90% confluence in a 10 cm dish. After washing three times with PBS solution (pH 7.4) and incubating in 3 mL of serum free *α*‐MEM medium containing 10 µg mL^−1^ Cytochalasin B (Solarbio, Beijing, China) for 30 min at 37 °C, morphological changes of the cells were dynamically observed under fluorescence microscopy (Essen, Incucyte ZOOM System). Then the cells and formed CMVs were detached by incubation with 0.25% w/v trypsin and 0.01% w/v ethylenediaminetetraacetic acid (EDTA) at 37 °C for 1 min, followed by trypsin inactivation via addition of the same volume of FBS. The mixture was transferred into a 15 mL centrifuge tube and agitated on a vortex for 30 s to separate cells and the CMVs. Then the mixture was centrifuged at 200 g for 5 min to remove cells and aggregated CMVs. The pelleted cells were discarded, while the supernatants were collected. After a second centrifugation at 2000*g* for 20 min, the pelleted CMVs were obtained. These CMVs were then re‐dispersed in the medium for further experiments or were stored in FBS + 10% v/v dimethyl sulfoxide at −20 °C .

### The Characterization of CMVs

The fluorescent‐labeled CMVs were obtained from the fluorescent‐labeled cells. The cells were stained with 10 µg mL^−1^ DiI (Solarbio) at 37 °C for 10 min, washed with PBS for three times, and subsequently stained with 10 µg mL^−1^ CFDA‐SE (carboxyfluorescein diacetate, succinimidyl ester, Solarbio) in serum‐free medium at 37 °C for 1 h. Then the CMVs were collected after separation from the parental cells and observed under laser scanning confocal microscopy (CLSM, Leica). The morphology and the structure of the CMVs were also observed under TEM. The size measurement (by DLS at room temperature) and *ζ*‐potential measurement of CMVs were performed by Malvern Zetasizer nano ZS.

### Cell Viability Assay

The cell viability of Raw264.7 cells was assessed using a Cell Counting Kit (CCK‐8, Dojindo Laboratories, Japan). 1.0 × 10^4^ cells per well were seeded into a 96‐well plate and incubated overnight. Then the cells were incubated with CMVs at different concentrations (the number ratios of cells to CMVs were ≈1:5; 1:10; 1:15) for 6, 12, and 24 h, respectively. Raw264.7 cells incubated without CMVs were used as controls. The CCK‐8 reagent was added to each well at determined time intervals, according to the manufacturer's protocol. Then the absorbance was determined at 450 nm by an enzyme‐linked immunological detector, after incubation for 120 min.

### Preparation of Engineered CMVs with High Expression of CXCR4

A PCR‐amplified gene fragment encoding CXCR4 was cut using restriction enzymes and cloned into a lentiviral vector (Gnenchem, Shanghai, China). The NCBI accession number for the CXCR4 gene sequence is NM_009911. 3T3 cells plated within 24‐well culture dishes were transfected with CXCR4 recombinant lentivirus at a multiplicity of infection (MOI) of 50. GFP expression was observed under fluorescence microscopy. 72 h after the transfection, there was peak fluorescence intensity, and the transfected cells were expanded for further experiments. CMVs with enriched membrane CXCR4 expression were prepared from lentivirus transfected cells using the previous method.

### Western Blot Analysis

The cells and CMVs were lysed in radioimmunoprecipitation assay buffer (Beyotime, China) containing a protease and phosphatase inhibitor cocktail (Thermo Fisher Scientific). After the proteins were quantified and denatured, samples were separated by 10% w/v sodium dodecyl sulfate‐polyacrylamide gel electrophoresis. The separated proteins were transferred to a polyvinylidene fluoride membrane at 300 mA for 60 min. Membranes were then blocked in Tris‐buffered saline containing Tween‐20 (TBST) and 5% (w/v) skimmed milk for 1 h. The western blot was carried out with the anti‐CXCR4 monoclonal antibody (1:1000, Abcam) and mouse anti‐GAPDH monoclonal antibody (1:5000, Abcam) at 4 °C overnight. Then the membranes were incubated with sheep anti‐mouse antibody (1:500, Beyotime) for 1 h. The blots were visualized using an ECL‐PLUS kit (Pierce). The relative expression of proteins was compared through band density.

### Incorporation of Curcumin into CXCR4‐CMVs and Release of Drugs

CXCR4/Cur‐CMVs were prepared by mixing CXCR4‐CMVs with curcumin in PBS at 37 °C for 20 min. The DiI‐labeled CXCR4‐CMVs was mixed with curcumin as described above and observed under CLSM (Leica) at OD_420_. The DiI‐labeled CXCR4‐CMVs without curcumin were utilized as controls. Then the *ζ*‐potential measurement of CMVs and CXCR4‐CMVs were determined by Malvern Zetasizer nano ZS. A stock solution of curcumin was diluted to a range from 0.15 to 2.5 mg L^−1^. Then the standard calibration curve was made by plotting the concentration of standard curcumin and fluorescent absorbance at 420 nm. To estimate the loading efficiency of curcumin into CXCR4‐CMVs, 10 µg CXCR4‐CMVs in 1 mL PBS was mixed with the same volume of curcumin at different concentrations (0, 0.2, 0.4, 0.8, 1.6 and 2.4 mg mL^−1^). After combining curcumin and CMVs, the CXCR4/Cur‐CMVs were centrifuged and re‐suspended. Then the concentration of curcumin in the suspended solution was calculated at OD_420_ based on the standard curve. Free curcumin and CXCR4/Cur‐CMVs were added to 1.5 mL PBS to achieve a final concentration of 20 µmol L^−1^ and incubated at 37 °C in the dark. Then 100 µL aliquots of each sample were collected to determine the concentration of curcumin every 30 min for 2.5 h at OD_420_. The starting concentrations of different samples were set at a relative value of 1.0. Graphs of the various data sets were then plotted, with the remaining percentages compared to the initial relative value (*n* = 3). For drug release assay in vitro, the curcumin released from CXCR4‐CMVs were evaluated by a dialysis method. 115 µg CXCR4/Cur‐CMVs dispersed in 1 mL PBS (PH = 7.4) were transferred to a dialysis bag (MWCO: 3000) and then placed in 20 mL PBS with 0.5% v/v Tween‐80 and 20% v/v ethanol. These were then placed in water at 37 °C with constant stirring for 24 h. At predetermined timepoints, 1 mL of receptor medium was removed and replaced with the same volume of receptor medium, so as to keep the total volume equal. The concentrations of curcumin in the released solution were calculated based on reading off a standard curve at OD_420_.

### Anti‐Inflammation Activity of CMVs Curcumin

5.0 × 10^5^ RAW 264.7 cells were plated in 6‐well culture dishes and incubated overnight. The cells were treated with free curcumin, Cur‐CMVs, and CXCR4/Cur‐CMVs for 1 h to achieve a final concentration of 15 µmol L^−1^ and then stimulated with LPS (500 ng mL^−1^, Sigma, USA) for 6 h. The cells treated with PBS for 1 h and then LPS for 6 h were set as controls. The untreated cells were set as the blank group. The cell supernatant was collected and centrifuged at 3000 rpm min^−1^ for 15 min at 4 °C and then IL‐6, IL‐1*β*, and TNF‐*α* expression levels in the supernatants were detected using a double antibody sandwich ELISA kit (Beijing gersion Bio‐Technology Co., Ltd.), according to the manufacturer's instructions. Then the total RNA from cells in the four groups was extracted and reversely transcribed into cDNA with RNA extract and PrimeScript RT reagent kit (Takara Co. Japan), respectively. The cDNA thus obtained was further amplified via a real‐time quantitative‐polymerase chain (q‐PCR) reaction under the following amplification conditions: 95 °C for 30 s, following by 39 cycles of 95 °C for 5 s and 60 °C for 30 s. Optical 96‐well reaction plates and optical adhesive films (Thermo Fisher Scientific) were used for PCR. A 20 µL volume of the PCR mixture, which included 8 µL of FastStart Universal SYBR Green Master Mix (Rox), 10 µL of RNase‐free water, 1 µL of template cDNA, and 1 µL of primer, was loaded into each well. PCR amplification was conducted with the following cycling parameters: 15 min at 95 °C (heat activation step), followed by 40 cycles of 15 s at 95 °C and 1 h at 60 °C. Data were analyzed using QuantStudio Design & Analysis Desktop Software (Thermo Fisher Scientific). IL‐6, IL‐1*β*, and TNF‐*α* gene expression levels were calculated according to the cycle threshold (Ct) values relative to the endogenous housekeeping control gene (GAPDH). Differences in gene expression levels among different groups were statistically analyzed. The primer sequences are presented in **Table** **1**. Glyceraldehyde‐3‐phosphate dehydrogenase (GAPDH) served as the internal control.

**Table 1:** Primer sequences utilized for quantitative real‐time PCR analysis

Target gene	Forward sequence (5′–3′)	Reverse sequence (5′–3′)
IL‐6	TTCTTGGGACTGATGCTGGTG	GCCATTGCACAACTCTTTTCTC
IL‐1*β*	TCAAATCTCGCAGCAGCACATC	CGTCACACACCAGCAGGTTATC
TNF‐*α*	CCACCACGCTCTTCTGTCTACTG	GCTCCTCCACTTGGTGGTTTGT
iNOS	AACATCAGGTCGGCCATCACT	CAGAGGCAGCACATCAAAGC
Arg‐1	TTACAAGACAGGGCTCCTTTCAG	GCTTATGGTTACCCTCCCGTTG
CXCR4	AAGAAGCTAAGGAGCATGACGG	GGCGTGGACAATAGCGAGGT
GAPDH	CCTCGTCCCGTAGACAATG	TGAGGTCGAAGGGGTCGT

### In Vitro Antimicrobial Test

Antimicrobial activity of curcumin was evaluated by assaying its inhibitory effects on the growth of *Enterococcus faecalis* (ATCC 29212). *E. faecalis* was cultured at 37 °C on nutrient agar plates (Solarbio) under aerobic conditions. Individual colonies of the bacteria were seeded into 10 mL of brain heart infusion (BHI, Solarbio) broth and grown overnight at 37 °C with agitation. Then 2 × 100 µL aliquots of 1 × 10^6^ CFU mL^−1^ bacteria suspension was added to the 96‐well flat‐bottomed microtiter plate (Corning, USA). To evaluate the antimicrobial activity, the experimental groups containing *E. faecalis* were subjected to 100 µL of i) PBS, ii) curcumin, iii) Cur‐CMVs, iv) CXCR4/Cur‐CMVs, 1.25% w/v NaClO for 1 h at 37 °C, respectively (*n* = 3). The curcumin dosage was 2 mg mL^−1^ in all drug‐containing groups. After different treatments, 10 µL of each well was cultured on BHI agar plates and incubated at 37 °C for 24 h. The number of CFUs mL^−1^ was determined according to a method described by Miles et al.^[^ ^35^ ^]^

### Transwell Migration Assay

Evaluation of CMVs migration was performed using a 24‐well transwell chamber of 3 µm pore size (Costar, USA). 5 µg DiI‐stained CMVs and CXCR4‐CMVs were mixed with 100 µL collagen I (Corning) and seeded into the upper chamber. After the formation of collagen I for 30 min, 100 ng mL^−1^ CXCL12 (Peprotech, USA) dispersed in 300 µL PBS was placed into the lower chamber, while PBS without CXCL12 served as controls (*n* = 3). Then the chambers were incubated for 1 h at 37 °C within a humidified atmosphere with 5% CO_2_. PBS in the lower chamber was collected, mixed well, and placed into a glass bottom dish (Cellvis, USA) for laser scanning confocal microscopy analysis. The 3D images of CMVs distribution were observed. The counting of CMVs at every layer of the scanning field was analyzed via Image J‐2.0.0.

### Ulcerative Colitis Model

All animal experiments were conducted with the approval of the Animal Care and Use Committee of Peking University (Approval number: LA2018215). Forty 6–8 weeks old BALB/c male mice (weight: 22 ± 2 g) were ordered and fed in a pathogen‐free facility. All animals were adapted for 1 week before the animal surgeries. Mice were provided with 3% w/v DSS (MP Biomedicals) dissolved in drinking water for 7 days to induce acute intestinal inflammation. Mice given normal water were set as the blank group.

### In Vivo Distribution

On the 7th day after induction of colitis, 18 colitis mice were randomly assigned into three groups: i) control group (PBS injected only), ii) Cur‐CMVs group, and iii) CXCR4/Cur‐CMVs group. DiI‐labeled Cur‐CMVs and CXCR4/Cur‐CMVs dispersed in 100 µL PBS were injected into mice via tail veins. The dosage of curcumin injected was 10 mg kg^−1^. After 12 h, the injected mice were culled to harvest the intestinal tissue. Then the samples were observed under CLSM and the fluorescence intensity and fluorescence volume of DiI were analyzed by LAS X (Leica Co.).

### The Biodistribution In Vivo

After 18 mice mentioned above were sacrificed, the major organs (brain, heart, liver, spleen, lung, and kidney) were collected from each mouse for detection of the fluorescence signal under CLSM.

### Intestinal Inflammation Treatment

On the 5th day following the induction of colitis, 24 colitis mice were randomly assigned into four groups: i) control group (PBS injected only), ii) free curcumin group, iii) Cur‐CMVs group, and iv) CXCR4/Cur‐CMVs group. The dosage of injected curcumin was 10 mg kg^−1^ in all drug‐containing groups. Drugs were injected from the 5th day to the 9th day via the tail vein once a day. 6 mice provided with normal water through all these procedures were set as the blank group. The body weight and DAI score were recorded throughout the experiment. The DAI scores were defined as follows: weight loss = 0 (no loss), 1 (1% to 5%), 2 (5% to 10%), 3 (10% to 20%), and 4 (>20%); ii) stool consistency or diarrhea = 0 (normal), 1 (slightly soft), 2 (loose), 3 (unformed/mild diarrhea), and 4 (severe diarrhea); iii) bleeding = 0 (no blood), 1 (faint positive fecal occult blood), 2 (certain positive fecal occult blood), 3 (moderate rectal bleeding), and 4 (gross bleeding). The DAI was the average of the scores for weight loss, stool consistency or diarrhea, and bleeding. On the 10th day, 30 mice were culled to harvest the entire colon and the colon lengths were recorded. 100 mg of colon tissues were collected and homogenized. Then total RNA was extracted using TRIzol, and cDNA was synthesized by RT‐PCR kit according to the manufacturer's instructions. IL‐6 and IL‐1*β* gene expression levels were analyzed according to the Ct values relative to the blank group gene (GAPDH). For histopathological analysis, the colon tissues from the same position of each colon were fixed in paraffin and stained with H&E. The tissue sections were observed under an optical microscope. The histopathological scores were graded from 0 to 3 by the sum of the scores of epithelial cell damage, crypt destruction, and infiltration of inflammatory cells according to a previous study.^[^ ^36^ ^]^

### Animal Toxicity Test

For safety evaluation of Cur‐CMVs and CXCR4/Cur‐CMVs, the major organs (heart, liver, spleen, lung, and kidney) of the mice injected with PBS, Cur‐CMVs, and CXCR4/Cur‐CMVs, as mentioned above, were collected from each individual mouse, and subjected to histological processing for H&E staining.

### Induction of AP in Mice and Injection of Cell Membrane Vesicles

Forty 6–8 weeks old BALB/c male mice (weight: 22 ± 2 g) were ordered and fed in a pathogen‐free facility. All animals were adapted for 1 week before surgeries were performed. 6–8 weeks male Balb/c mice were anesthetized by intraperitoneal injection of 4% w/v chloral hydrate. A quarter round bur was used to drill the left mandibular first molars.

### In Vivo Distribution

After 21 days of pulp exposure to the oral environment, 18 mice were randomly assigned into three groups and received different treatments via intravenous tail injections: i) PBS group (PBS injected only), ii) CMVs group, and iii) CXCR4‐CMVs group. Since fluorescence of cFDA‐SE was uniform and stable, cFDA‐SE‐labeled CMVs and CXCR4‐CMVs at 5 mg kg^−1^ in 100 µL PBS were injected. 24 h after injection, the bilateral mandibular first molar and surrounding bones were collected and fixed with 4% w/v paraformaldehyde. Then the samples were decalcified for frozen slicing and immunofluorescence of CXCR4/CXCL12.

### AP Inflammation Inhibition

After 21 days of pulp exposure, the bilateral mandibular first molars and surrounding bones of 6 mice were collected for H&E staining and immunohistochemistry for detection of IL‐6, IL‐1*β*, and TNF‐*α*, so to verify the construction of the periodontitis apical model; 6 untreated mice were set as the controls. Additionally, 24 mice were randomly assigned into four groups and received different treatments via intravenous tail injections: i) PBS group (PBS injected only), ii) Cur‐CMVs group, and iii) CXCR4/Cur‐CMVs group. The curcumin dosage was 10 mg kg^−1^ in all the drug‐containing groups. After being injected twice daily for 8 days, the left mandibular first molars and surrounding bones were collected and fixed with 4% w/v paraformaldehyde. Then the samples were decalcified for H&E staining and immunohistochemistry to detect expression of IL‐6, IL‐1*β*, and TNF‐*α*.

### Hematoxylin and Eosin Staining

The samples were collected and fixed in 4% w/v paraformaldehyde, decalcified in 10% w/v EDTA solution followed by dehydration, then embedded in paraffin, and sectioned at 4 mm (Leica Instruments GmbH, Hubloch, Germany). Sections were evaluated histologically by hematoxylin‐eosin staining according to the manufacturer's protocol. The stained sections were then observed under light microscopy (Olympus, Tokyo, Japan).

### Micro‐Computed Tomography Analysis

The bilateral mandibular sections were collected and the soft tissues were removed. Then the samples were scanned using a micro‐computed tomographic system (GANTRY‐ STD CT 3121; Siemens, Knoxville, TN) with 80 kV, 500 mA, a pixel size of 33.658 mm, a camera exposure time of 1500 ms, and 360 rotations around the vertical axis with a 1 rotation step. The aluminum filter was set at 0 mm during the scans. Raw data obtained at the scanning stage were reconstructed using the Inveon Research Workplace 4.2 software (Siemens).

### Immunofluorescence and Immunohistochemistry

The samples were embedded into optimum cutting temperature (OCT) compound and frozen at −80 °C . Then the frozen tissues were cut into 5 µm thick sections for immunohistochemical analysis. The frozen sections were incubated with rabbit anti‐CXCR4, rabbit anti‐CXCL12 (ZEN, BIO) overnight at 4 °C . The secondary 594‐donkey anti‐rabbit antibody (abcam, ab150076) was next incubated with the sample. The sections were then observed under CLSM (Leica).

Paraffin‐embedded mandibular sections were submitted to immunohistochemistry analysis. The samples were incubated with rabbit anti‐IL‐6 antibody, rabbit anti‐IL‐1*β* antibody, and rabbit anti‐TNF alpha antibody (ZEN, BIO) overnight at 4 °C . Then the sections were incubated with secondary anti‐rabbit antibody (Servicebio), and immersed in a DBA solution according to the manufacture's instruction. The percentage areas of positive inflammatory cytokine staining were analyzed using Image J 2.0.0 software. The method of area percentage analysis was as follows: 5 unit areas of apical tissues were selected randomly from each section and the mean value of all sections in one group was calculated as the area percentage value.

### Statistical Analysis

Statistical differences among the experimental groups were evaluated with one‐way analysis of variance on ranks test. Statistical differences between the two groups were evaluated by unpaired Student's *t*‐test. All statistical analyses were performed with PRISM software, version 7.0. A P value < 0.05 was considered statistically significant. Numerical results were presented as mean ± SD.

## Conflict of Interest

The authors declare no conflict of interest.

## Supporting information

Supporting Information

## Data Availability

Research data are not shared.
