# Supplementary material for: Cell Membrane Vesicles with Enriched CXCR4 Display Enhances Their Targeted Delivery as Drug Carriers to Inflammatory Sites
Source: Adv Sci (Weinh). 2021 Oct 23;8(23):2101562. doi: 10.1002/advs.202101562 (PMC8655180; doi:10.1002/advs.202101562)
Supplement: Supplementary file 1 — Supporting Information [file ADVS-8-2101562-s001.pdf]

ADVANCED SCIENCE
Open Access

Supporting Information

for *Adv. Sci.*, DOI: 10.1002/advs.202101562

Cell membrane vesicles with enriched CXCR4 display enhances their targeted delivery as drug carriers to inflammatory sites

Dandan Wang, Shengjie Jiang, Fengyi Zhang, Siqin Ma, Boon Chin Heng, Yuanyuan Wang, Junxia Zhu, Mingming Xu, Ying He, Yan Wei, Xuehui Zhang, Bin Xia and Xuliang Deng**

Supporting Information

Cell membrane vesicles with enriched CXCR4 display enhances their targeted delivery as drug carriers to inflammatory sites

Dandan Wang, Shengjie Jiang, Fengyi Zhang, Siqin Ma, Boon Chin Heng, Yuanyuan Wang, Junxia Zhu, Mingming Xu, Ying He, Yan Wei, Xuehui Zhang, Bin Xia and Xuliang Deng**

Table of contents

Supplementary Figure 1: The fibrous framework changes of MC-3T3 cells after treatment with Cytochalasin B (CB).

Supplementary Figure 2: The size distribution of CMVs.

Supplementary Figure 3: Assessment of CMVs toxicity.

Supplementary Figure 4: The transfection efficiency of lentivirus vector encoding *CXCR4/GFP* chimeric protein.

Supplementary Figure 5: Representative TEM images of CXCR4-CMVs.

Supplementary Figure 6: Surface Z-potential of CMVs, Cur-CMVs, CXCR4-CMVs, and CXCR4/Cur-CMVs.

Supplementary Figure 7: Representative CLSM images of CMVs and CXCR4-CMVs without curcumin co-culture.

Supplementary Figure 8: Markedly increased solubility and stability of curcumin after encapsulation within CXCR4-CMVs *in vitro*.

Supplementary Figure 9: The standard curve of curcumin.

Supplementary Figure 10: Representative TEM images of Cur-CMVs (a) and CXCR4/Cur-CMVs (b).

Supplementary Figure 11: Morphological changes of polarized macrophages after different treatments in the three groups.

Supplementary Figure 12: Effects of free curcumin, Cur-CMVs, CXCR/Cur-CMVs, CXCR4/Cur-CMVs and 1.25% (w/v) NaClO against Enterococcus faecalis.

Supplementary Figure 13: The targeting effects of CXCR4/Cur-CMVs in the DSS-induced ulcerative colitis model.

Supplementary Figure 14： Live fluorescence tracking of CMVs, CXCR4-CMVs and curcumin within inflammatory colonic tissues.

Supplementary Figure 15: Representative photographs of the colon in five groups: Blank, DSS, Cur, Cur-CMVs, and CXCR4/Cur-CMVs.

Supplementary Figure 16: IL-6 and IL-1β expression in colon tissues of mice following different treatments.

Supplementary Figure 17: The biodistribution of Cur-CMVs and CXCR4/Cur-CMVs in a colitis mice model.

Supplementary Figure 18. The toxicity of Cur-CMVs and CXCR4/Cur-CMVs *in vivo*.

Supplementary Figure 19: Establishment of the periapical periodontitis model.

Supplementary Figure 20: CXCL12 and cFDA-SE labeled CXCR4/Cur-CMVs expression within the periapical lesions and normal periapical tissues.

Supplementary Figure 21: CXCR4 and cFDA-SE labeled CXCR4/Cur-CMVs expression within normal periapical tissues.

Supplementary Figure 22: The percentage of areas stained positive for IL-6 were analyzed with Image J 2.0.0 software.

Supplementary Figure 23: The immunohistochemical staining of mandibular first molar in each group.

Supplementary Figure 24: Representative immunohistochemical staining images of Runx2 expression in the mandibular first molar of each group.

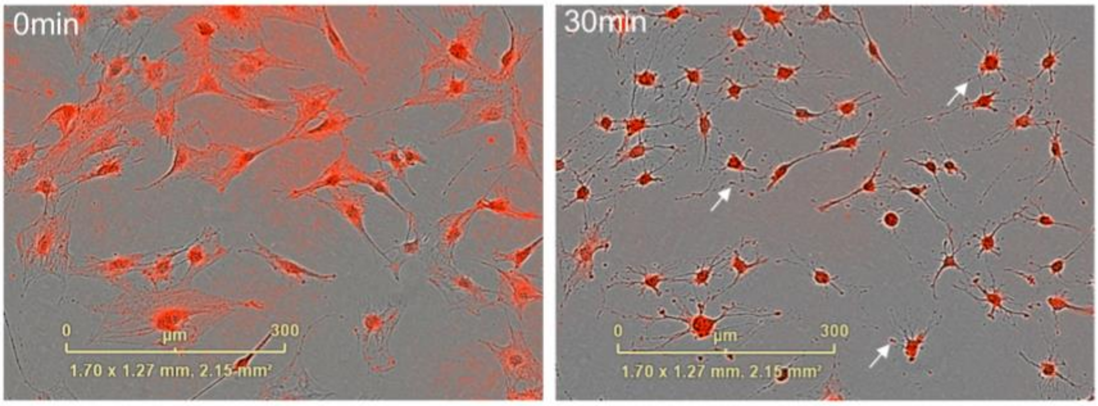

Supplementary Figure 1: The fibrous framework change of MC-3T3 cells after treatment with Cytochalasin B (CB). DiI-labeled MC-3T3 cells were dynamically observed under fluorescence microscopy after treatment with CB for 30 min. The white arrows mark the cell vesicles surrounding the cells.

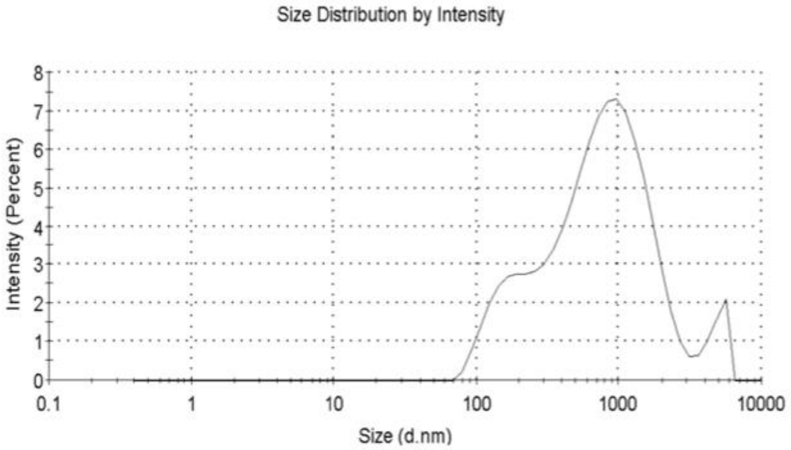

	Size (d.nm...	% Intensity:	St Dev (d.n...
**Peak 1:**	827.4	94.0	594.0
**Peak 2:**	4642	6.0	850.4
**Peak 3:**	0.000	0.0	0.000

Supplementary Figure 2: The size distribution of CMVs. The peak analyzed by DLS showed that the average size of CMVs was approximately 824.7 nm (d, diameter) and their intensity was about 94%.

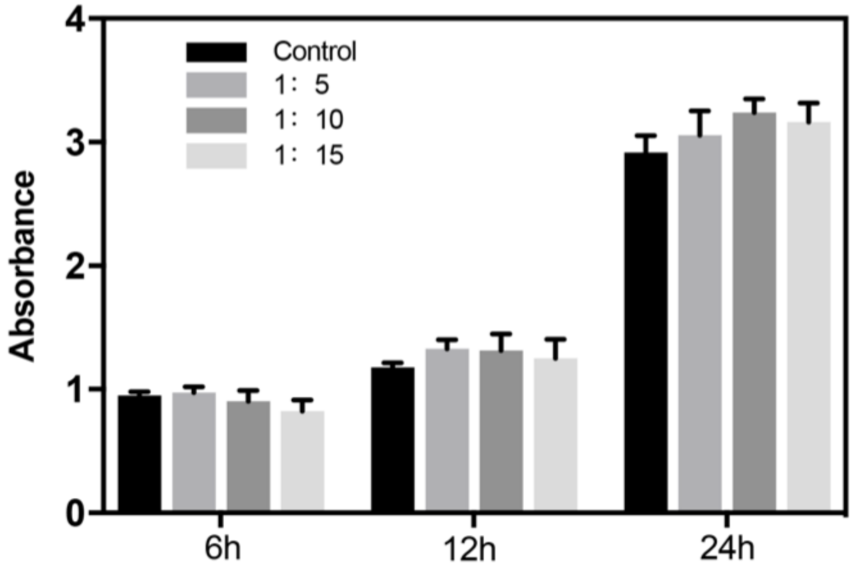

Supplementary Figure 3: Assessment of CMVs toxicity. The cell viability assay of RAW264.7 cells incubated with different concentrations of CMVs (the number ratios of RAW264.7 cells to CMVs were approximately 1:5; 1:10; 1:15) for 6 h, 12 h, 24 h. Results showed no significant difference among the groups (n=5).

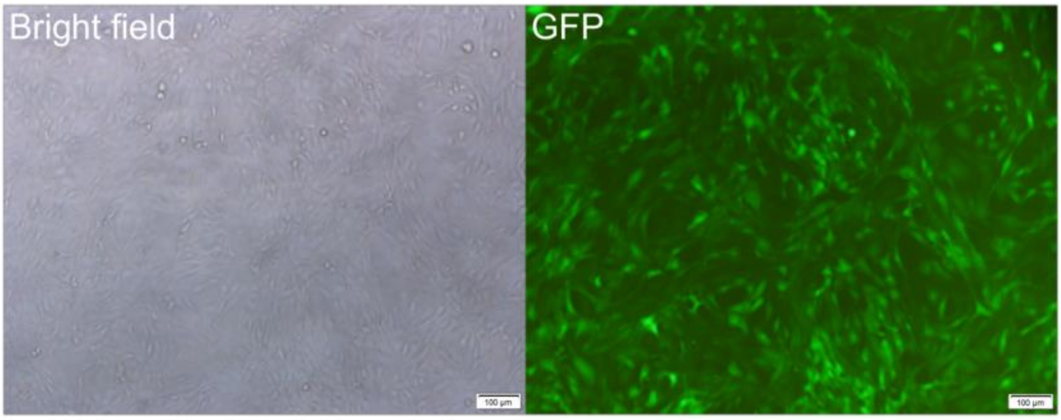

Supplementary Figure 4: The transfection efficiency of lentivirus vector encoding *CXCR4/GFP* chimeric protein. Green fluorescent protein (GFP) signals in 3T3 cells showed a high rate of transfection after recombinant lentiviral transfection (MOI of 50) for 72 h.

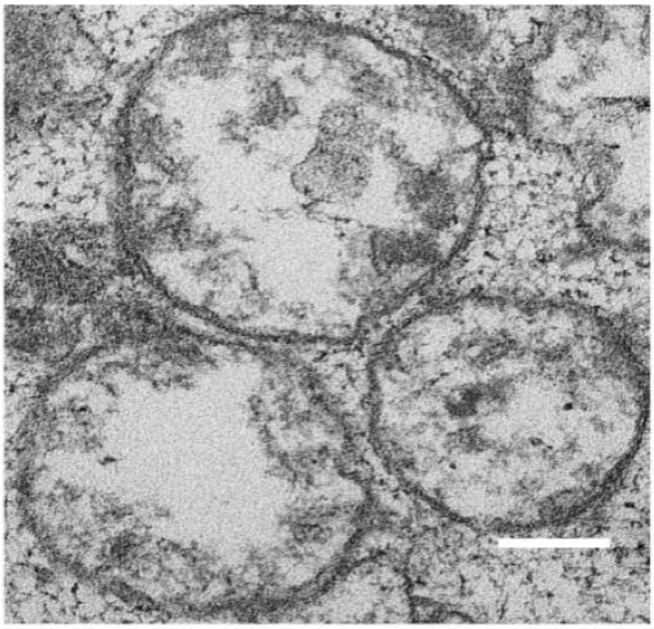

Supplementary Figure 5: Representative TEM images of CXCR4-CMVs. The CMVs with overexpressed CXCR4 maintained membrane integrity. The scale bar = 300 nm.

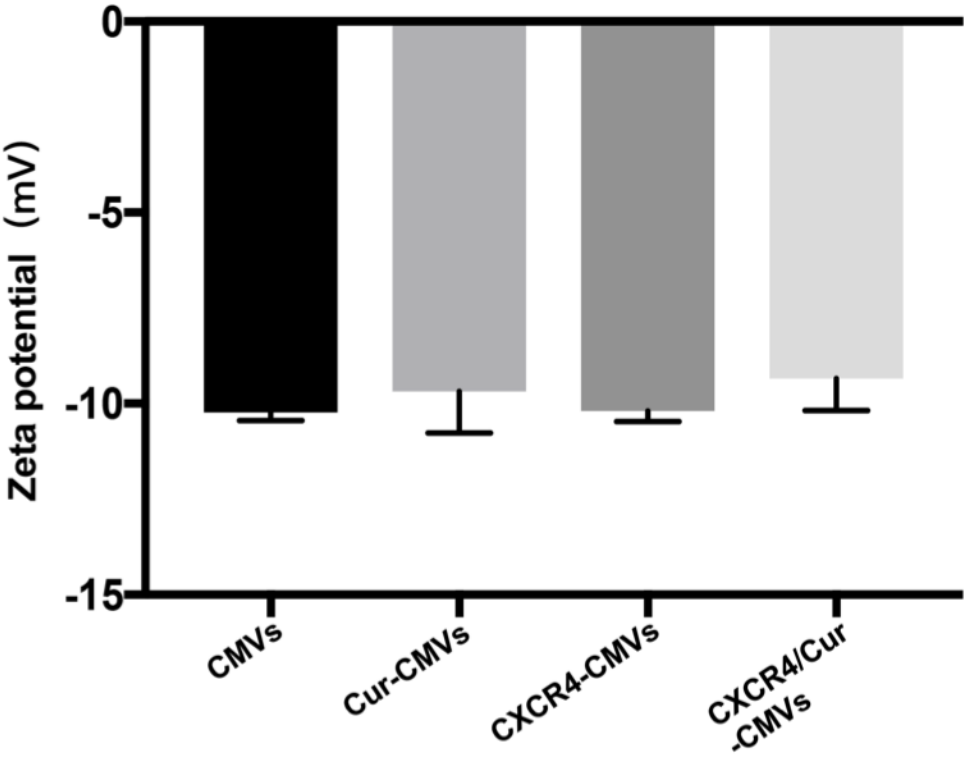

Supplementary Figure 6: Surface Z-potential of CMVs, Cur-CMVs, CXCR4-CMVs, and CXCR4/Cur-CMVs. Results showed no significant differences among groups (P＞0.05, n=3).

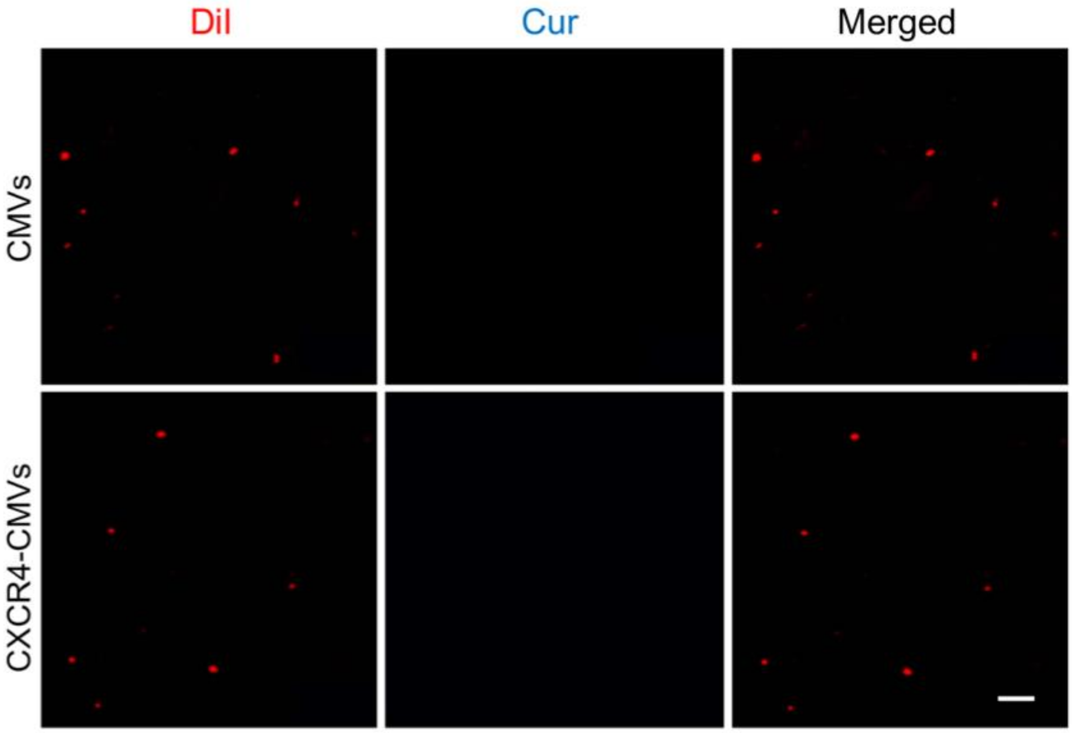

Supplementary Figure 7: Representative CLSM images of CMVs and CXCR4-CMVs without curcumin co-culture. Images showed DiI-labeled CMVs and CXCR4-CMVs (red). The scale bar = 5 μm.

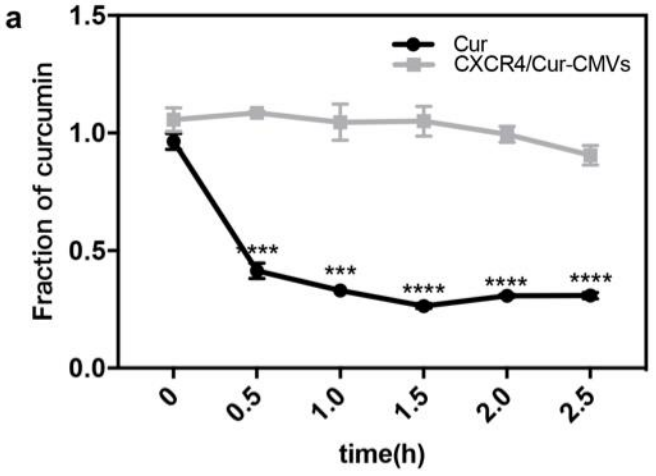

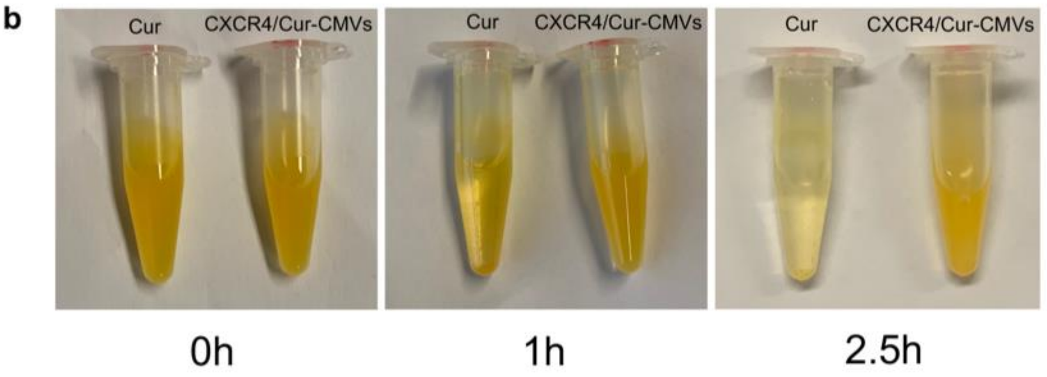

Supplementary Figure 8: Markedly increased solubility and stability of curcumin after encapsulation within CXCR4-CMVs *in vitro*. (a) Free curcumin and CXCR4/Cur-CMVs were added to 1 ml of PBS to achieve a final concentration of 10 μmol/L, which were then incubated in the dark at 37℃ and in a humidified atmosphere containing 5% CO_2. The OD_{420} value was determined at 0, 30, 60, 90, 120 and 150 min. The results showed that only 30% of free curcumin remained in PBS, while approximately 90% remained after loading in CXCR4-CMVs after 150 minutes (***p＜0.001, ****P＜0.0001, n=3). **(b)** The color of curcumin encapsulated into CXCR4-CMVs slightly lightened, while the color of free curcumin in PBS lightened rapidly within 2.5 h.

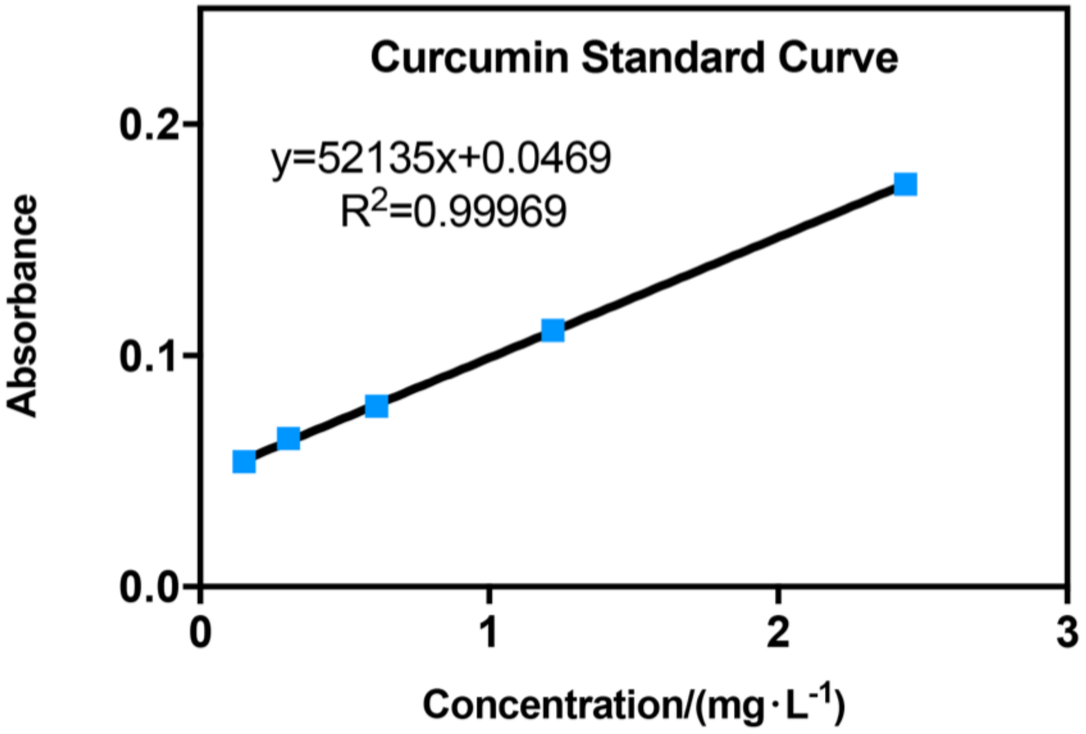

Supplementary Figure 9: The standard curve of curcumin. The calculated curcumin standard curve was fitted as y=52135x+0.0469, R^2=0.99969, where y represents the absorbance and x represents the drug concentration.

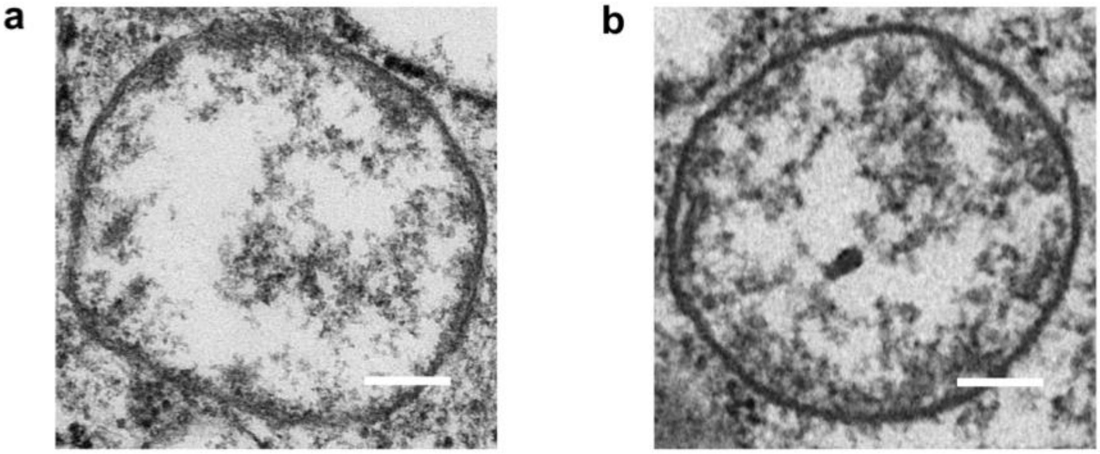

Supplementary Figure 10: Representative TEM images of Cur-CMVs (a) and CXCR4/Cur-CMVs (b). The results showed intact membrane structure of CMVs and CXCR4-CMVs after encapsulation with curcumin respectively. Scale bars = 200 nm.

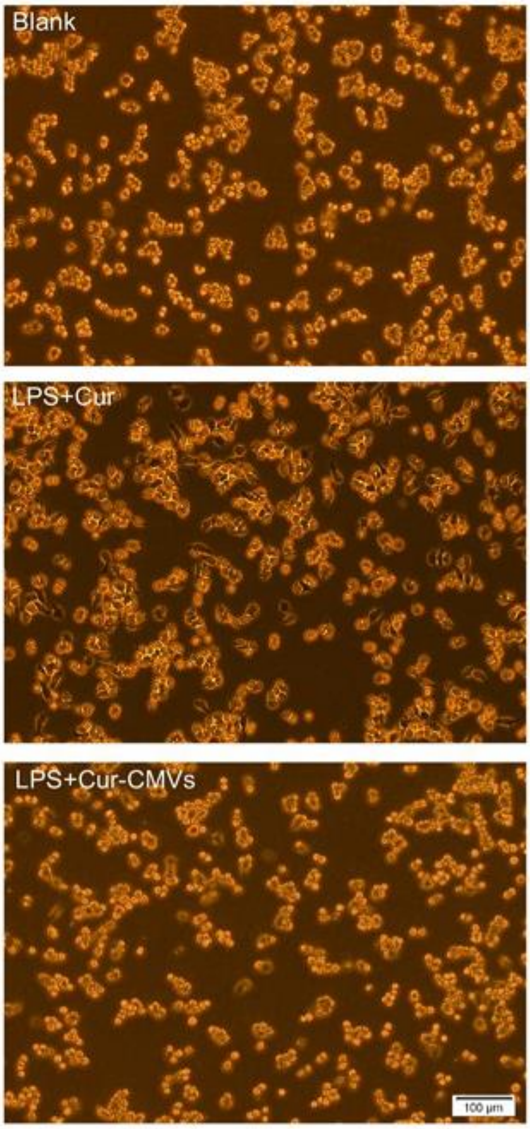

Supplementary Figure 11: Morphological changes of polarized macrophages after different treatments in the three groups. Macrophages in LPS plus Cur, LPS plus Cur-CMVs groups were treated with free curcumin and Cur-CMVs for 1 hour respectively before induction with LPS for another 6 hours. Macrophages in blank, LPS plus Cur-CMVs showed similar morphology of round shape whereas apparent “synapses” of macrophage were exhibited in LPS plus free curcumin group.

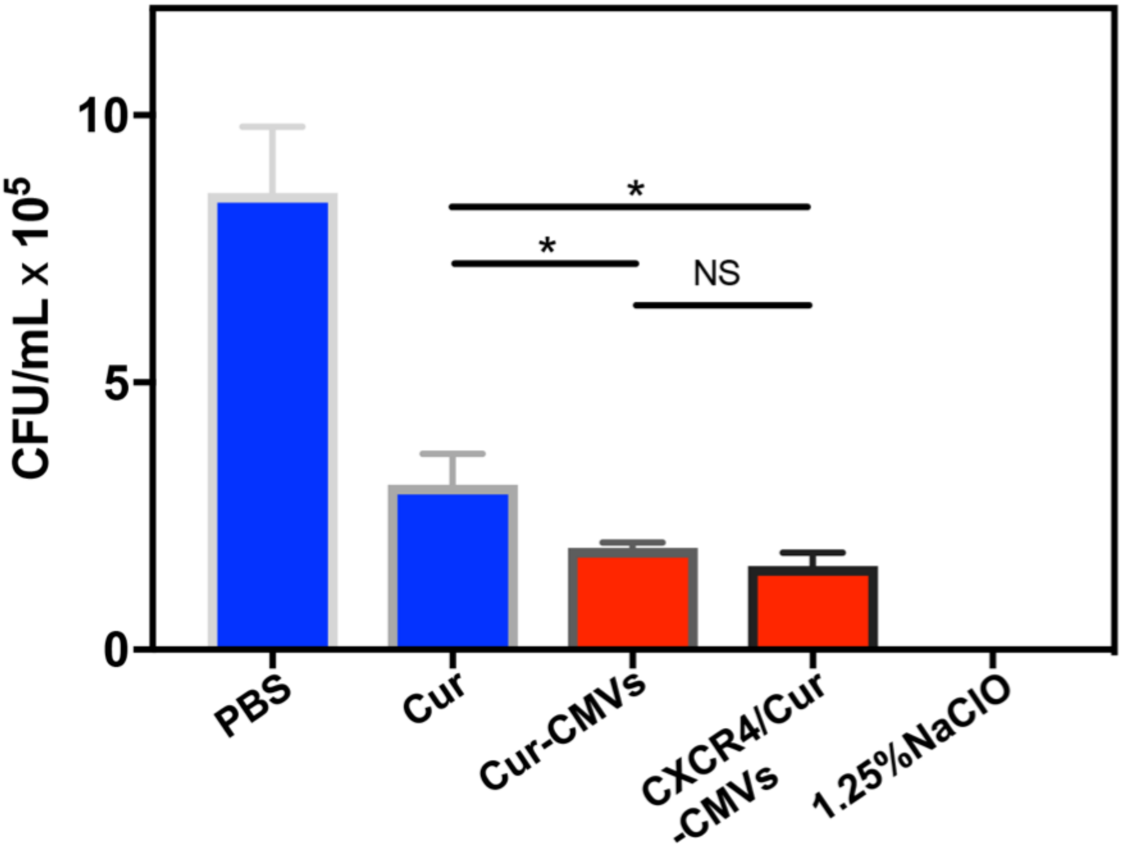

Supplementary Figure 12: Effects of free curcumin, Cur-CMVs, CXCR/Cur-CMVs and 1.25% (w/v) NaClO against Enterococcus faecalis. CFU/mL: colony forming units per milliliter. Control: PBS. The results showed that the curcumin loaded into CMVs and CXCR4-CMVs inhibited the growth of E. faecalis more effectively than the free curcumin (*P<0.05, n=3).

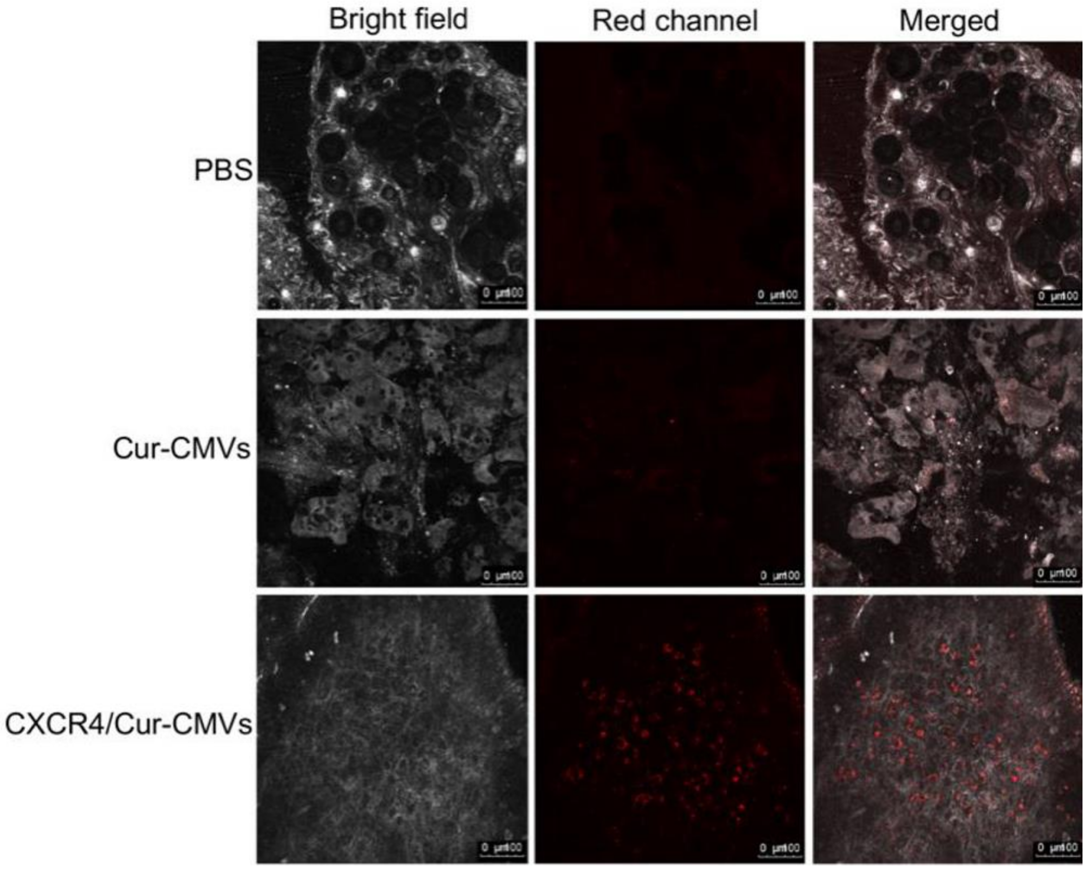

Supplementary Figure 13: The targeting effects of CXCR4/Cur-CMVs in the DSS-induced ulcerative colitis model. The results showed significant red fluorescence staining after DiI-labeled CXCR4/Cur-CMVs injection, while only weak red fluorescent signals were observed in the colonic tissues of the control and Cur-CMVs groups (n=6).

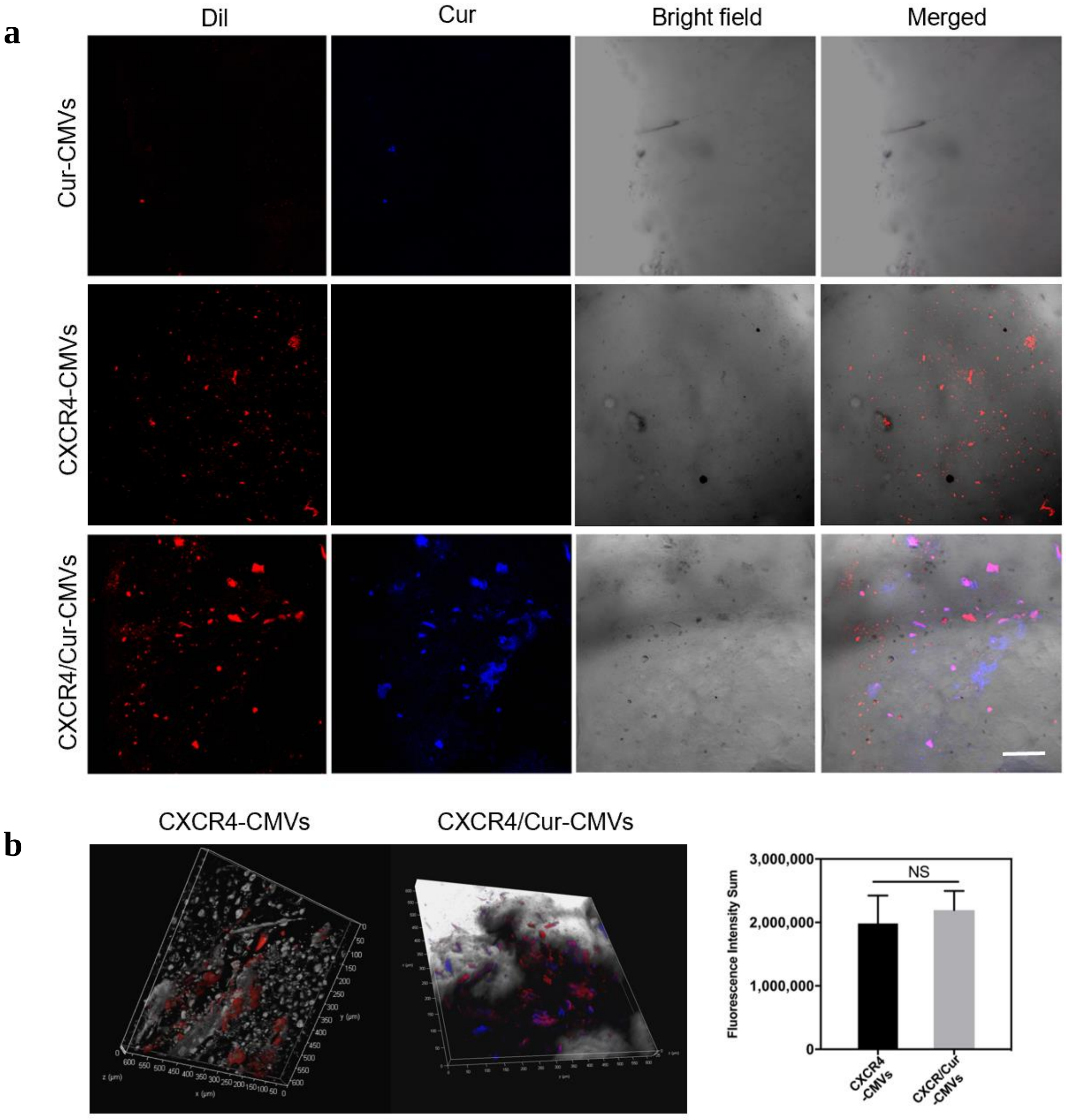

Supplementary Figure 14 : Live fluorescence tracking of CMVs, CXCR4-CMVs and curcumin within inflammatory colonic tissues. **(a)** The intestinal tissues of colitis mice injected with CXCR4-CMVs and CXCR4/Cur-CMVs showed much higher DiI fluorescence expression (red) and curcumin auto-fluorescence expression (blue), as compared to colitis mice injected with Cur-CMVs (n=6). **(b)** There was no significant difference in fluorescence intensity between CXCR4-CMVs and CXCR4/Cur-CMVs(n=6). The scale bar = 25 μm.

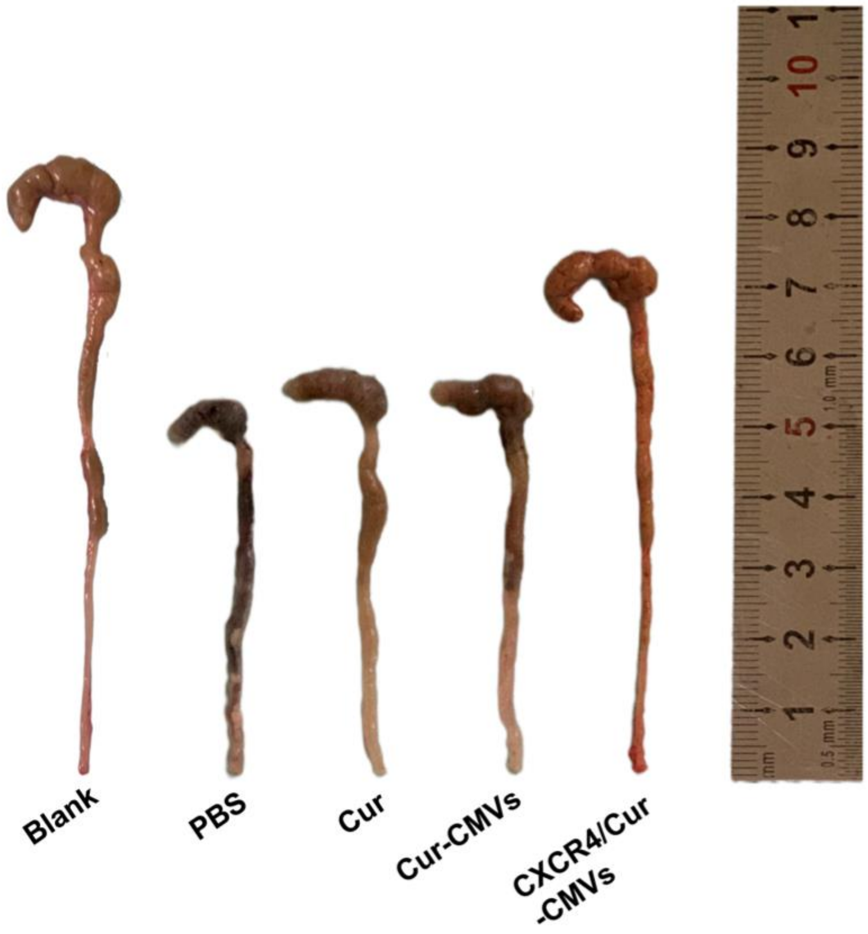

Supplementary Figure 15: Representative photographs of the colon in five groups: Blank, DSS, Cur, Cur-CMVs, and CXCR4/Cur-CMVs. The colon length in the CXCR4/Cur-CMVs group was only slightly shorter than the blank group, whereas the mice in the DSS, free curcumin and Cur-CMVs injection groups had drastically shortened colon length (n = 6).

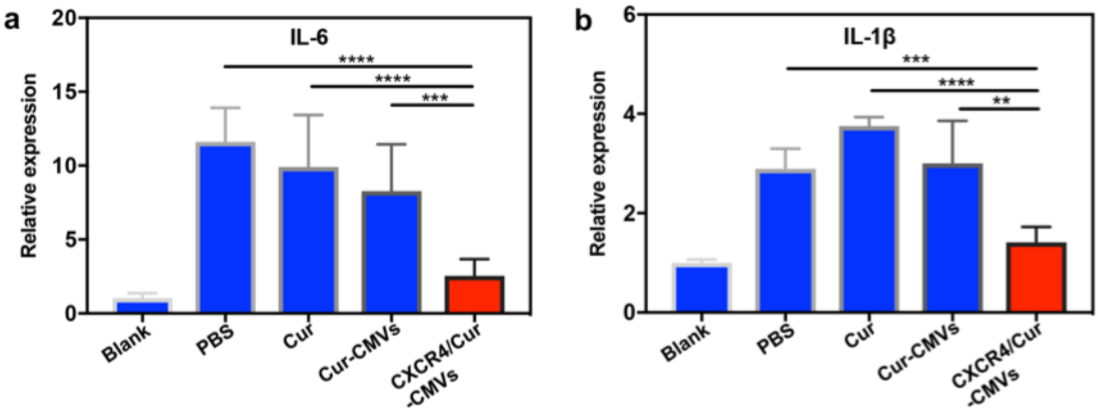

Supplementary Figure 16: IL-6 and IL-1β expression in colon tissues of mice following different treatments. The mRNA level of IL-6 (**a**) and IL-1β (**b**) of the colonic tissues in the CXCR4/Cur -CMVs group were significantly lower than the control and Cur-CMVs groups, and slightly higher than the blank group (**P＜0.01， ***P＜0.001,****P＜0.0001,n=6).

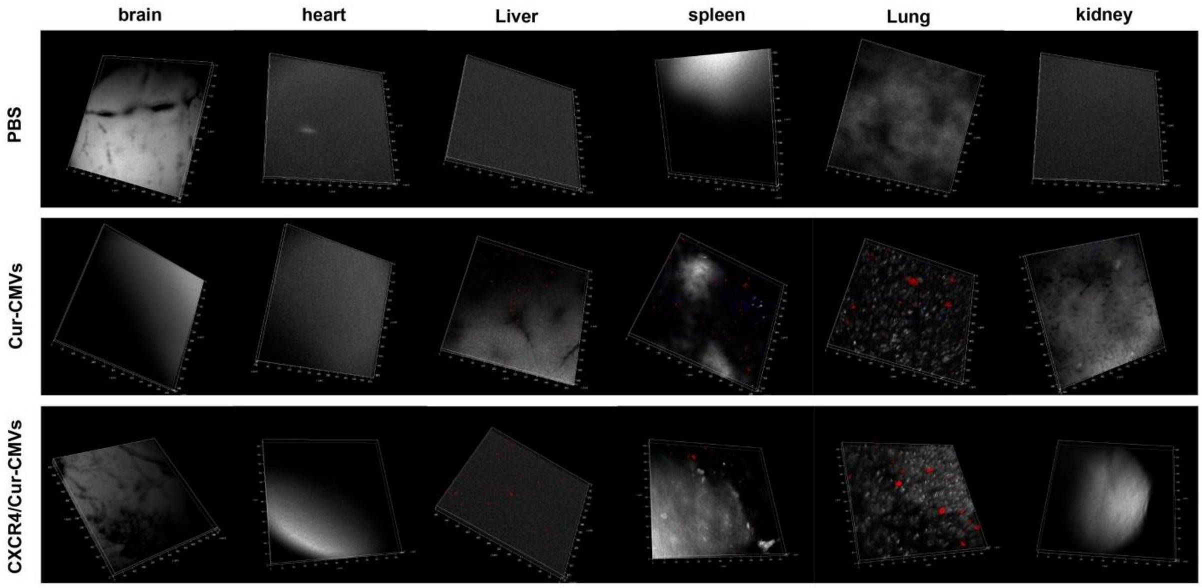

Supplementary Figure 17: The biodistribution of Cur-CMVs and CXCR4/Cur-CMVs in a colitis mice model. The fluorescence imaging shows the biodistribution of Dil-labeled Cur-CMVs and CXCR4/Cur-CMVs (red) in the major organs.

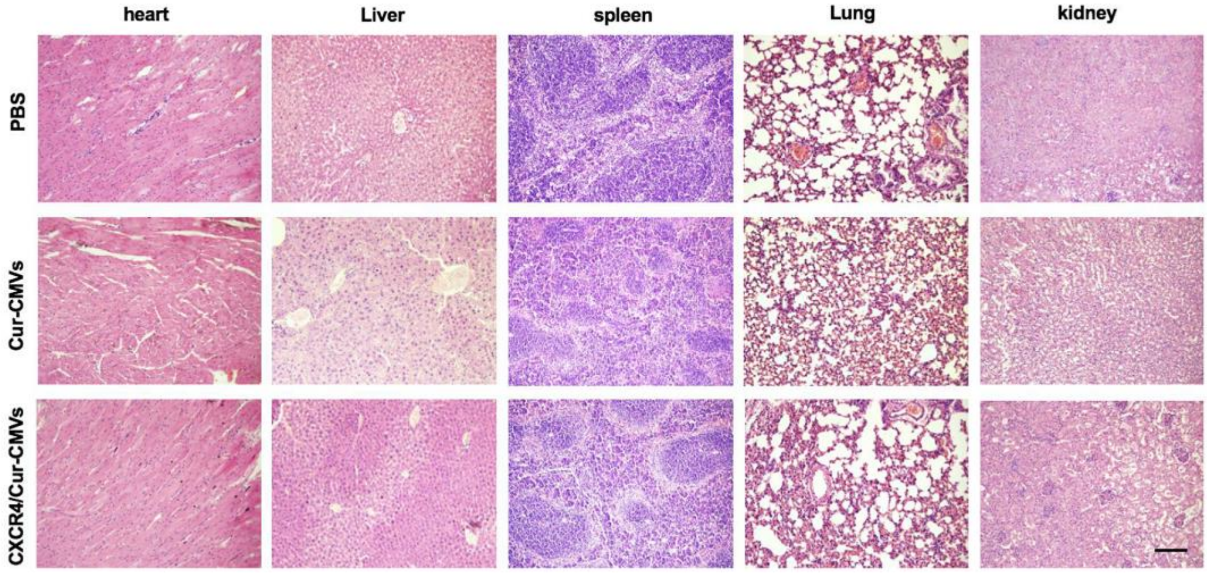

Supplementary Figure 18. The toxicity of Cur-CMVs and CXCR4/Cur-CMVs *in vivo*. H&E staining of heart, liver, spleen, lung and kidney in the colitis model after different treatments. The scale bar = 100 μm.

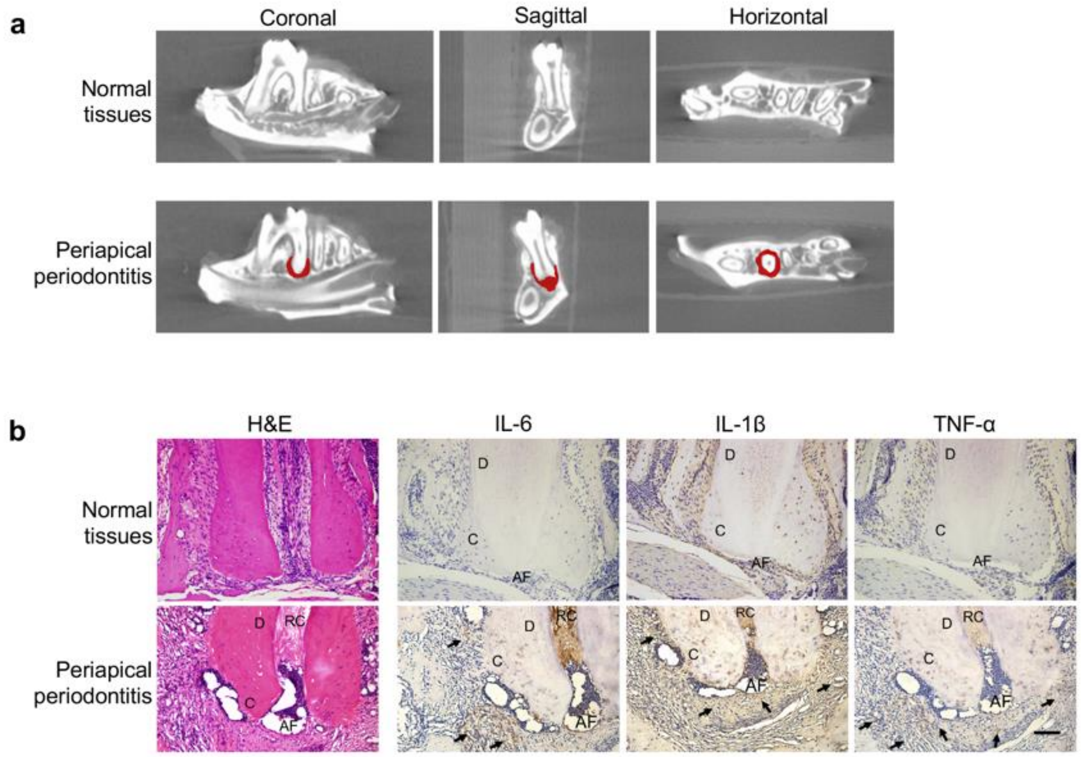

Supplementary Figure 19: Establishment of the periapical periodontitis model. After pulp exposure for 21 days, the mandibular first molars were collected for micro-CT scanning, H&E and immunohistochemical staining. The untreated teeth on the opposite side of the mandibular sections were set as controls. Representative images of Micro-CT (**a**), histological and immunohistochemical images (**b**) of the infected mandibular first molar showed the establishment of the apical periodontitis model in mice. The red zones showed bone resorption in the infected apical areas. The D denotes dentin; the C denotes cementum; the AF denotes apical foramen; the RC denotes root canal. The black arrows mark the immunohistochemical positive areas. The scale bar = 200 μm.

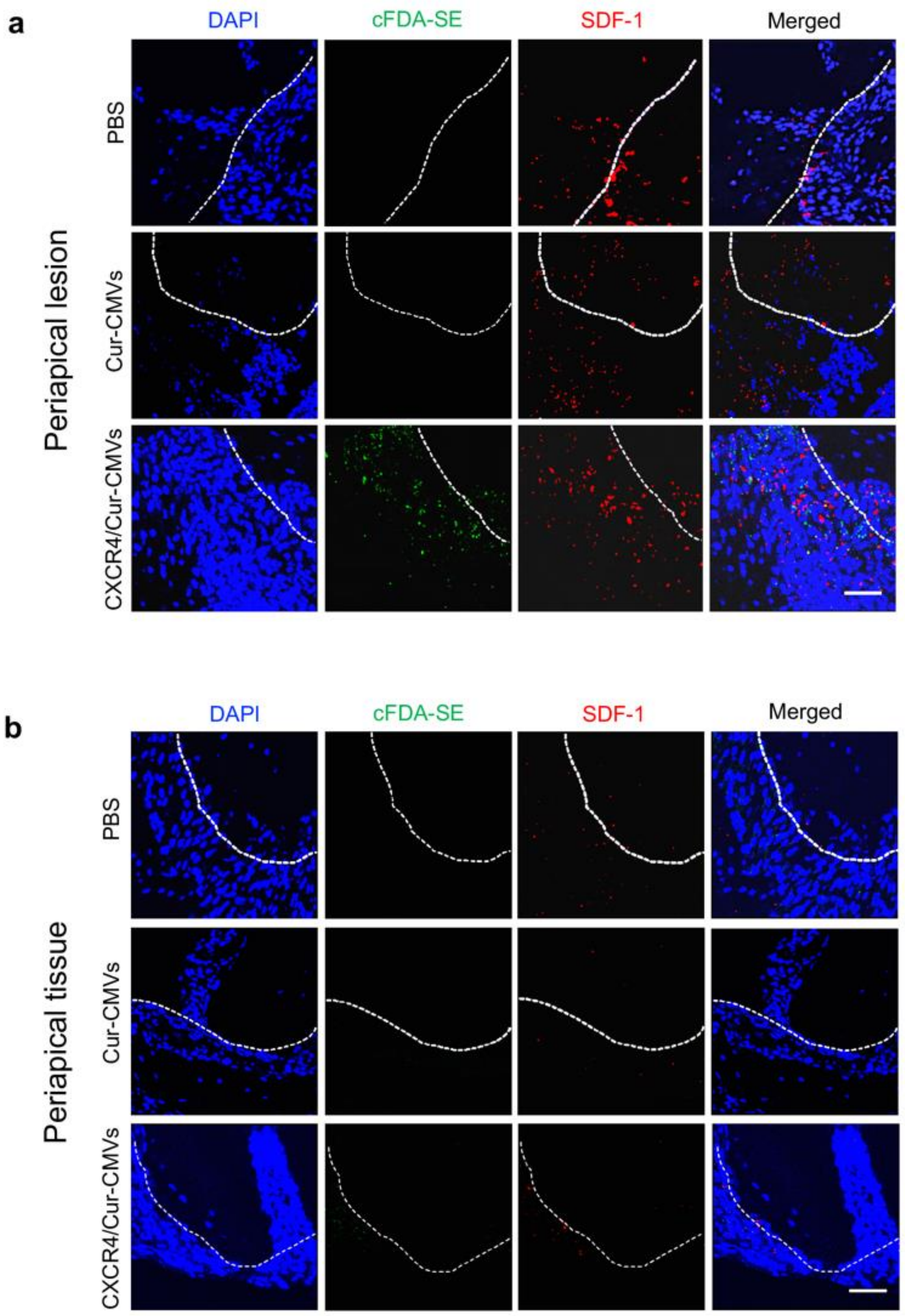

Supplementary Figure 20: CXCL12 and cFDA-SE labeled CXCR4/Cur-CMVs expression within the periapical lesions and normal periapical tissues. (a) After pulp exposure for 21 days, there was higher expression of CXCL12 (red) in the periapical lesions, while cFDA-SE labeled CXCR4/Cur-CMVs (green) aggregated in the corresponding areas. **(b)** The fluorescence images of normal periapical tissues were set as controls. Scale bars = 50 μm.

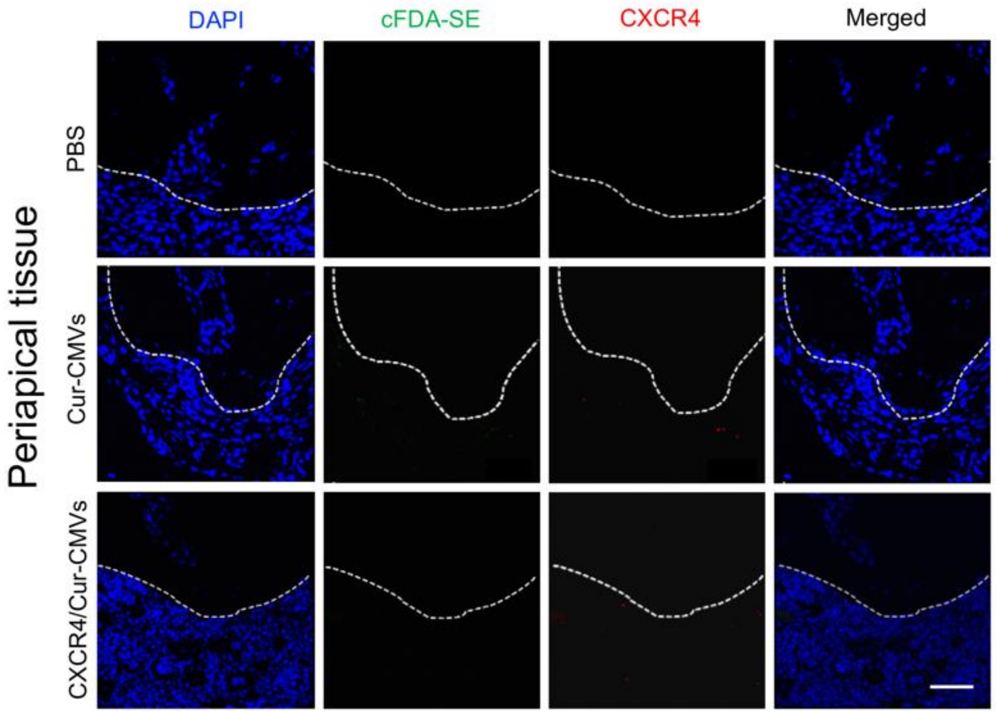

Supplementary Figure 21: CXCR4 and cFDA-SE labeled CXCR4/Cur-CMVs expression within normal periapical tissues. The fluorescence images of normal periapical tissues showed fairly low CXCR4 and cFDA-SE expression. The scale bar = 50 μm.

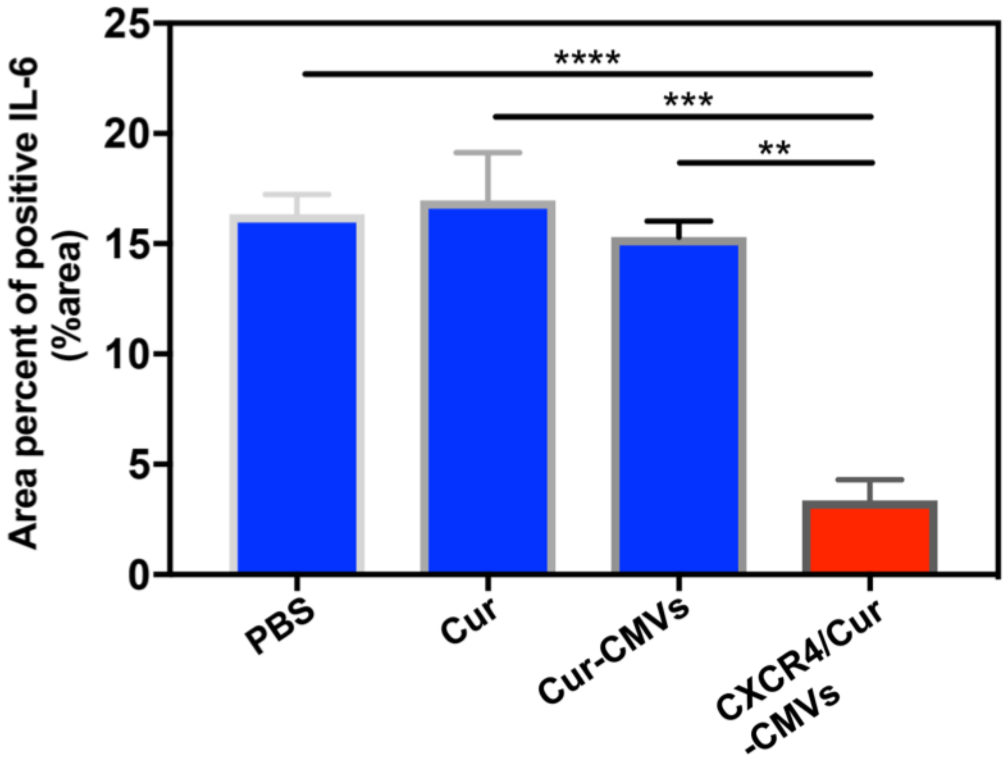

Supplementary Figure 22: The percentage of areas stained positive for IL-6 were analyzed with Image J 2.0.0 software. After injection of CXCR4/Cur-CMVs, the IL-6 secretion within infected apical tissues markedly decreased compared to the control, free curcumin and Cur-CMVs injection groups (**P＜0.01，***P＜0.001,****P＜0.0001,n=6).

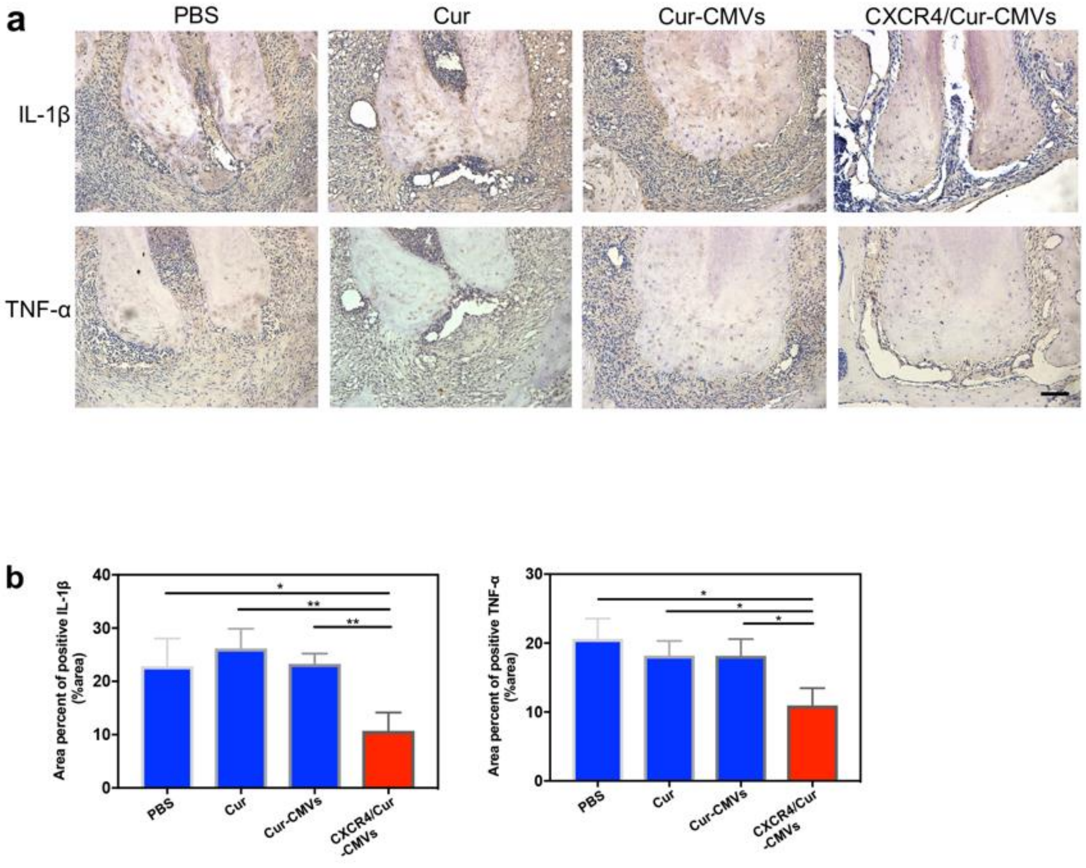

Supplementary Figure 23: The immunohistochemical staining of the mandibular first molar in each group. (a) Representative immunohistochemical staining images of mandibular first molar in mice with different treatment: (i) Control group (PBS injected only), (ii) free curcumin group, (iii) Cur-CMVs group, and (iv) CXCR4/Cur-CMVs group. The black arrows mark the immunohistochemical positive areas. The scale bar = 200 μm. **(b)** The percentage positive staining areas of IL-1β and TNF-α were analyzed with Image J 2.0.0 software (*P＜0.05, **P＜0.01, n=6).

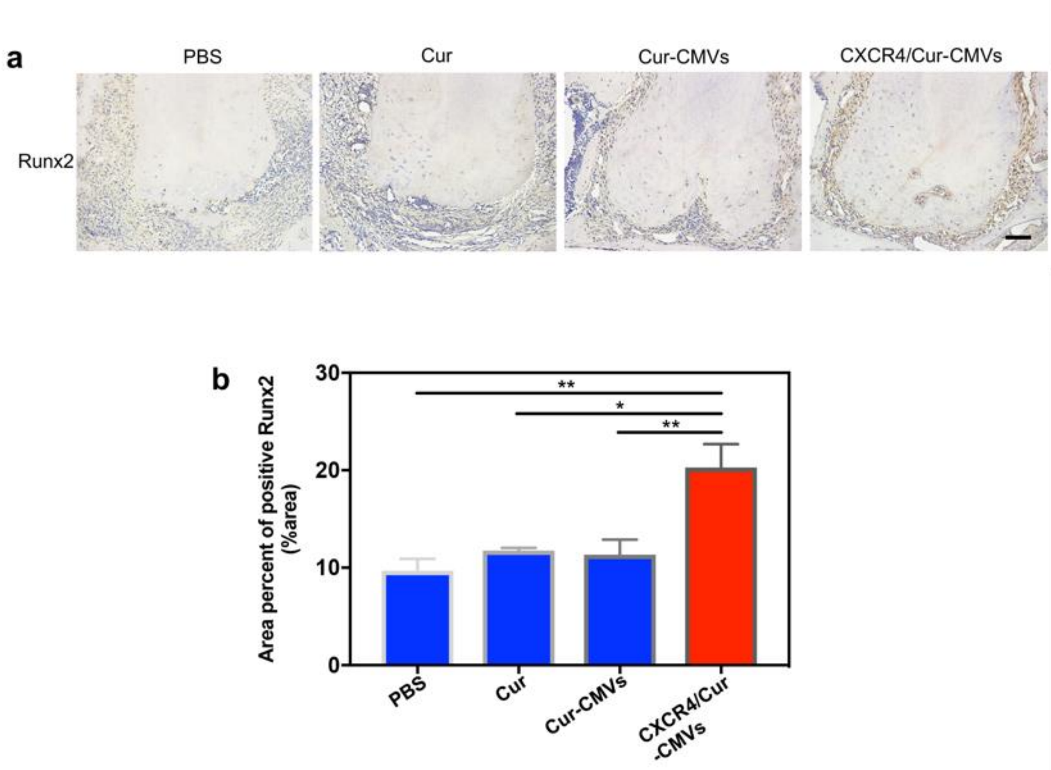

Supplementary Figure 24: Representative immunohistochemical staining images of Runx2 expression in the mandibular first molar of each group. (a) Representative immunohistochemical staining images of Runx2 expression in mandibular first molar after different treatments: (i) Control group (PBS injected only), (ii) free curcumin group, (iii) Cur-CMVs, and (iv) CXCR4/Cur-CMVs. The scale bar = 200 μm. **(b)** The percentages of positive stained areas of Runx2 were analyzed with Image J 2.0.0 software (*P＜0.05, **P＜0.01, n=6).
